# Melatonin and Selenium Enhance Strawberry Resistance to *Botrytis cinerea* via Integrated Transcriptome–Metabolome Analysis

**DOI:** 10.3390/foods15183291

**Published:** 2026-09-17

**Authors:** Yanru Zhou, Huawei Zang, Haochen Du, Farooq Muhammad Raza, Panpan Wang, Linxi Yuan, Zhi-Qing Lin, Li Wang, Miao Li

**Affiliations:** 1 Key Laboratory of Agri-Products Quality and Biosafety of Ministry of Education, Anhui Province Engineering Laboratory for Green Pesticide Development and Application, Anhui Province Key Laboratory of Crops Integrated Pest Management, Key Laboratory of Biology and Sustainable Management of Plant Diseases and Pests of Anhui Higher Education Institutes, Anhui Province Key Laboratory of Functional Agriculture and Functional Food, School of Plant Protection, Anhui Agricultural University, Hefei 230036, China; 2 School of Biomedical and Environmental Engineering, Hefei Institute of Technology, Hefei 238076, China; 3 School of Earth and Space Sciences, University of Science and Technology of China, Hefei 230026, China; 4 Department of Health and Environmental Sciences, Xi’an Jiaotong-Liverpool University, Suzhou 215123, China; 5 Department of Environmental Sciences, Southern Illinois University Edwardsville, Edwardsville, IL 62026, USA; 6 Key Laboratory of Jianghuai Agricultural Product Fine Processing and Resource Utilization of Ministry of Agriculture and Rural Affairs, School of Tea and Food Science & Technology, Anhui Agricultural University, Hefei 230036, China

**Keywords:** strawberry fruits, *Botrytis cinerea*, melatonin, selenium, disease resistance, multi-omics analysis

## Abstract

This study fills a critical knowledge gap by elucidating the mechanisms of melatonin (MT) and selenium (Se) in enhancing strawberry resistance to *Botrytis cinerea* through integrated physiological, biochemical, transcriptomic, and metabolomic analyses, providing an eco-friendly strategy for postharvest disease management. Combined MT (0.5 mmol/L) and sodium selenite (25 mg/L) exhibited a strong inhibitory effect on *B. cinerea*, reducing colony diameter to 1.05 cm with an inhibition rate of 93.67%, compared with MT (14.08%) alone or sodium selenite alone (42.87%). MT+Se treatment significantly reduced gray mold incidence in strawberry fruits to <15% at 96 h, versus >75% in controls, while maintaining higher soluble sugar (13.25 vs. 7.01 mg/g) and protein (0.286 vs. 0.186 mg/g) contents. Moreover, MT+Se attenuated oxidative damage by lowering malondialdehyde (MDA) levels (7.47 vs. 11.63 nmol/g FW) and enhancing antioxidant enzyme activities (e.g., POD: 12.918 vs. 8.411 U/g FW). Integrated transcriptomic–metabolomic analyses revealed that MT+Se upregulated antioxidant enzyme genes (SOD, CAT, POD, and APX), resistance-related genes, and transcription factors, while enhancing flavonoid and phenylpropanoid biosynthesis and promoting secondary metabolite accumulation (e.g., epicatechin, naringenin chalcone). Overall, combined MT+Se treatment enhanced strawberry resistance to *B. cinerea* by boosting antioxidant defense and resistance pathways, offering an eco-friendly strategy for postharvest disease control.

## 1. Introduction

Strawberries (*Fragaria* × *ananassa* Duch.) are highly susceptible to both biotic and abiotic stresses during growth, development, transportation, and storage [1,2,3,4]. *Botrytis cinerea*, the causal agent of gray mold, is recognized as one of the top ten fungal plant pathogens in molecular plant pathology. *B. cinerea* severely threatens strawberry production by exploiting high-humidity conditions to infect flowers, fruits, leaves, and stems, causing rot, necrosis, and up to 50% yield loss [3,5,6]. The disease primarily affects leaves, petioles, flowers, and fruits [7]. Postharvest fragility and varietal susceptibility—such as the ‘Hongyan’ cultivar in the Yangtze River Delta—further amplify its economic impact [8,9]. Additionally, ripe strawberries carrying *B. cinerea* conidia remain susceptible to latent infections during postharvest transport and storage. Chemical control remains the main method for strawberry gray mold management. Currently used effective fungicides include procymidone, iprodione, azoxystrobin, carbendazim, isoprothiolane, pyraclostrobin, and fluopyram [10]. However, the long-term and excessive use of these chemicals has led to the development of fungicide resistance in *B. cinerea*, causing some fungicides to lose effectiveness, while also creating irreversible environmental pollution [11,12]. Therefore, there is an urgent need to develop eco-friendly disease-control strategies that can directly inhibit pathogen growth and/or enhance host defense responses by regulating antioxidant, metabolic, and signaling pathways.

Melatonin (MT), a naturally occurring indoleamine and plant signaling molecule, plays a crucial role in plant growth and stress responses [13,14]. It promotes root development, regulates flowering and fruiting, delays senescence, enhances antioxidant capacity, and mitigates oxidative stress [15]. Melatonin also exhibits antifungal properties, with its derivative Mt-23 targeting fungal mitogen-activated protein kinases (Mps1), effectively reducing diseases such as rice blast, Fusarium wilt, and tomato gray mold [16]. Furthermore, MT acts as a signalling molecule, modulating phytohormone-related gene expression, including those associated with auxins, gibberellins, abscisic acid, salicylic acid, cytokinins, jasmonic acid, and ethylene [17]. Although melatonin has been shown to mitigate the impact of gray mold disease in certain plants by enhancing antioxidative defense and secondary metabolism, its role in strawberry remains only partially explored, leaving a critical research gap regarding its precise mechanisms and efficacy.

Selenium (Se), an essential trace element for humans and animals, also benefits higher plants, and can enhance tolerance to environmental stresses such as salinity, drought, and extreme temperatures while boosting antioxidant defense systems to mitigate oxidative damage [18,19]. Selenium has been shown to directly inhibit pathogens like *Alternaria tenuis* and *Fusarium* sp., and its exogenous application can induce the synthesis of secondary metabolites [20,21]. In potato late blight, exogenous selenium treatment upregulated phenylpropanoid pathway genes, increasing phenolic compound production and reducing disease severity [22]. The combined effects of MT and Se on strawberry resistance to gray mold remain insufficiently understood, particularly regarding their direct antifungal activity and potential induction of host defense responses.

Enhanced antioxidant enzyme activities have been associated with increased kiwifruit resistance to *Alternaria alternata*, while the accumulation of phenolic compounds and flavonoids has also been implicated in fruit defense against fungal pathogens [23,24]. Melatonin, recently identified as a novel plant hormone, and selenium, an essential micronutrient, have both been shown to enhance plant resistance to pathogens and abiotic stress, making them promising eco-friendly agents for disease control. However, their combined role in controlling strawberry gray mold and the underlying mechanisms, particularly their direct antifungal effects and their ability to enhance host resistance, remain poorly understood. Therefore, this study seeks to elucidate the direct antifungal effects of melatonin and selenium, alone and in combination, against *B. cinerea*, while determining their capacity to enhance host resistance, suppress gray mold infection, and maintain strawberry fruit quality. By integrating transcriptomic and metabolomic profiling, we aim to elucidate the molecular responses associated with MT+Se-enhanced host resistance, particularly those related to antioxidant defense and secondary metabolism. This study therefore provides a theoretical basis for understanding the resistance-enhancing effects of MT+Se and its potential application in postharvest gray mold control in strawberry fruits.

## 2. Materials and Methods

### 2.1. Pathogen, Plant, Chemicals

Strawberry fruits (*Fragaria* × *ananassa* ‘Red Face’) at 80% maturity were harvested during the strawberry production season from a strawberry garden in Changfeng County (Hefei, China). Fruits of uniform size and maturity, without visible physical damage or microbial infection, were selected from the same harvest batch. The harvested fruits were transported to the laboratory on the day of harvest under conditions that minimized mechanical damage and were immediately used for the experiments. The fruits were sterilised by immersion in 2% sodium hypochlorite for 2 min, rinsed twice with distilled water, and air-dried.

Na_2_SeO_3_ (97%) was purchased from Sinopharm Chemical Reagent Co. (Shanghai, China). Melatonin (98%) was purchased from Macklin Biochemical Technology Co. (Shanghai, China). All other reagents were of analytical grade and listed in the Supplementary Information.

The *B. cinerea* strain B05.10 used in this study was derived from the Institute of Botany, Chinese Academy of Sciences. It was placed on potato dextrose agar (PDA, Table S4) at 25 °C for incubation.

### 2.2. Cultivation of B. cinerea and Preparation of Conidial Suspension

The *B. cinerea* strain B05.10 was reactivated and subsequently transferred onto PDA plates. The inoculated plates were incubated at a constant temperature of 25 °C in a controlled incubator (MGC-250, Shanghai Yiheng Scientific Instrument Co., Ltd., Shanghai, China) until sufficient mycelial growth was obtained, after which they were maintained as stock cultures for experimental use.

For conidial suspension preparation, actively growing cultures of *B. cinerea* were incubated on PDA plates for approximately 15 days at 25 °C under dark conditions to allow robust sporulation. Following incubation, the aerial mycelia and conidia were gently scraped from the agar surface into a sterile 50 mL centrifuge tube containing sterile distilled water. To facilitate the dislodgement and uniform dispersion of conidia, two to three pre-sterilized stainless steel beads were added, and the suspension was subjected to vigorous agitation using a vortex mixer (ZWY-103B, Shanghai Zhicheng Analytical Instrument Manufacturing Co., Ltd., Shanghai, China). The resulting mixture was then passed through a double layer of sterile gauze to remove residual mycelial fragments, and the retained mycelia on the gauze were rinsed thoroughly with sterile distilled water to recover adherent conidia. The filtrate, containing dispersed conidia, was designated as the crude spore suspension.

The concentration of conidia in the suspension was quantified using a hemocytometer under a light microscope (CH20, Olympus Corporation, Tokyo, Japan). Based on the calculated density, the suspension was diluted with sterile distilled water to achieve a final standardized concentration of 1.0 × 10^6^ conidia per milliliter. Conidial suspensions were freshly prepared immediately prior to use in all subsequent assays to ensure viability and uniformity.

### 2.3. Effect of MT and Se on the Mycelial Growth of B. cinerea

To evaluate the antifungal activity of melatonin (MT) and sodium selenite against *B. cinerea*, mycelial plugs (0.6 cm in diameter) excised from actively growing colonies of uniform vigor were transferred to PDA media supplemented with different concentrations of sodium selenite (20, 30, 40, and 50 mg/L) or melatonin (0.01, 0.05, 0.1, 0.5, 1.0, 1.5, and 2.0 mmol/L). PDA plates amended with sterile distilled water served as the control (CK). All cultures were incubated at 25 °C under dark conditions until the radial growth of the control reached approximately two-thirds of the Petri dish diameter.

Mycelial expansion in each treatment was quantified by measuring colony diameters in two perpendicular directions, and the inhibition rate of fungal growth was calculated relative to the control group, expressed as a percentage (%). The concentration–response relationships were subjected to regression analysis using DPS statistical software (DPS 18.10). For sodium selenite, the EC_50_ was estimated from the fitted concentration–response regression model based on the inhibition rates obtained at 20, 30, 40, and 50 mg/L. The estimated EC_50_ was 25 mg/L, with a correlation coefficient of 0.98. The corresponding 95% confidence interval was calculated from the regression analysis. Based on these results, 25 mg/L sodium selenite and 0.5 mmol/L melatonin were selected for the subsequent combined-treatment experiments. Subsequently, a combined treatment experiment was conducted using the optimal concentrations of both agents to examine their combined inhibitory effect. Following exposure, the ultrastructural changes in *B. cinerea* cells, including alterations in cell wall integrity, plasma membrane permeability, were assessed using microscopic and biochemical analyses.

The fruits were randomly divided into two groups: one was treated with sterile water as the control (CK), and the other was treated with 25 mg/L sodium selenite and 0.5 mmol/L MT composite treatment (MT+Se). The fruits were also randomly assigned to the different sampling-time groups. A sterilized toothpick was used to make a 2 mm deep hole at the equatorial region of each strawberry fruit. After allowing the wound to dry (about 20 min), 10 µL of sterile water (CK) or the MT+Se solution was injected and allowed to absorb fully. A suspension of *B. cinerea* conidia (1 × 10^6^ spores/mL) was injected into the wound (10 µL per fruit). Treated fruits were stored at 22 °C and 85% relative humidity. Separate sets of fruits were used at each sampling time (0, 12, 24, 48, 72, and 96 h). For each treatment at each sampling time, 28 fruits were used per biological replicate, and the experiment was independently repeated three times (*n* = 3). The samples were collected at 0, 12, 24, 48, 72, and 96 h and immediately flash-frozen in liquid nitrogen for 10 min and stored at −80 °C for subsequent analyses.

### 2.4. SEM and Fluorescence Staining Analyses of B. cinerea

For scanning electron microscopy (SEM), *B. cinerea* mycelia were incubated in PDB containing MT and sodium selenite at 25 °C and 220 rpm for 5 h. The mycelia were collected by centrifugation at 5000 rpm for 10 min and fixed with 2.5% glutaraldehyde. Samples were washed three times with 0.1 mmol/L phosphate buffer (pH 7.0) for 15 min each and post-fixed with 1% osmium tetroxide for 2 h. After three additional washes with the same phosphate buffer, the samples were dehydrated through a graded ethanol series (30%, 50%, 70%, 80%, 90%, and 95%) for 15 min at each concentration, followed by two treatments with 100% ethanol for 20 min each. The samples were then treated with ethanol/isopentyl acetate (1:1, *v*/*v*) for 30 min, followed by pure isopentyl acetate for 1 h or overnight. After critical-point drying, the samples were observed using an S-3000N scanning electron microscope (Hitachi, Tokyo, Japan).

For propidium iodide (PI) staining, treated mycelia were washed three times with 0.1 mmol/L phosphate buffer (pH 7.0) by centrifugation at 12,000 rpm for 3 min. The mycelia were stained with 200 µL PI solution (100 µg/mL) and 20 µL DMSO for 15 min at room temperature in the dark. After staining, the mycelia were collected by centrifugation at 12,000 rpm for 3 min and washed three times with the same phosphate buffer. The stained mycelia were subsequently observed using a fluorescence microscope (CKX53, Olympus Corporation, Tokyo, Japan) with excitation wavelength of 400 nm. Three independent biological replicates were performed for each treatment.

For Calcofluor White staining, treated mycelia were incubated with 25 µg/L Calcofluor White solution (pH 10) for 15 min at room temperature. The stained mycelia were collected by centrifugation at 12,000 rpm for 3 min and washed three times with sterile water (pH 10) to remove excess stain. Chitin-associated fluorescence was subsequently observed using a fluorescence microscope. Three independent biological replicates were performed for each treatment.

### 2.5. Determination of Gray Mold Incidence, Soluble Sugar and Soluble Protein Content in Strawberry

Fruits showing visible gray mycelial growth were scored as diseased. Gray mold incidence (%) was calculated as the number of diseased fruits divided by the total number of fruits assessed × 100.

Soluble sugar content was measured using the 3,5-dinitrosalicylic acid (DNS) colorimetric method [25]. Fresh strawberry fruit tissue (0.5 g) was homogenized to obtain the extract. An aliquot of the extract was reacted with DNS reagent for color development, and the absorbance was measured spectrophotometrically at 540 nm. Soluble sugar content was calculated using a glucose standard curve and expressed as mg/g FW.

Soluble protein content was determined using the Coomassie Brilliant Blue method [26]. Fresh strawberry fruit tissue (0.5 g) was homogenized with quartz sand in 5 mL of 50 mmol/L phosphate buffer (pH 7.0), and the homogenate was centrifuged at 4000 rpm for 10 min at 4 °C. The resulting supernatant was reacted with Coomassie Brilliant Blue G-250 reagent, and absorbance was measured at 595 nm. Soluble protein content was calculated using bovine serum albumin (BSA) as the standard and expressed as mg/g FW.

### 2.6. Determination of MDA, ROS Levels and Antioxidant Enzyme Activities

Malondialdehyde (MDA) content was measured using the thiobarbituric acid (TBA) method [27]. Fresh strawberry fruit tissue (0.3 g) was thoroughly homogenized with quartz sand in 2 mL of 0.05 mol/L phosphate-buffered saline (PBS; pH 7.8). Subsequently, 5 mL of 0.5% TBA solution was added and thoroughly mixed. The mixture was heated in a boiling water bath for 10 min, rapidly cooled, and centrifuged at 3000 r/min for 15 min at 4 °C. Absorbance of the supernatants were determined at 532 nm and 600 nm. MDA content was calculated after correction for nonspecific absorbance at 600 nm and expressed as nmol/g FW.

Hydrogen peroxide (H_2_O_2_) content was assayed using a commercial assay kit (Nanjing Jiancheng Bioengineering Institute, Nanjing, China) according to the manufacturer’s instructions and expressed as µmol/L.

The superoxide anion (O_2−_) production rate was determined using the hydroxylamine oxidation method. The nitrite generated from the reaction between O_2−_ and hydroxylamine was quantified using a potassium nitrite standard curve, and the rate of O_2−_ production was expressed as µmol/min/g FW [28].

For antioxidant enzyme extraction, fresh strawberry fruit tissue (0.5 g) was homogenized in 5 mL of 50 mmol/L KH_2_PO_4_ buffer (pH 7.8) containing 0.2 mmol/L EDTA and 2% polyvinylpyrrolidone (PVP). The homogenate was centrifuged at 15,000 r/min for 30 min at 4 °C, and the resulting supernatant was used as the crude enzyme extract.

Superoxide dismutase (SOD) activity was measured by measuring the inhibition of photochemical reduction in nitroblue tetrazolium (NBT) [29]. Absorbance was measured spectrophotometrically at 560 nm. One unit (U) of SOD activity was defined as the amount of enzyme required to inhibit 50% of NBT photoreduction under the assay conditions, and SOD activity was expressed as U/g FW.

Catalase (CAT) activity was determined by monitoring the decomposition of H_2_O_2_ at 240 nm [29]. The reaction mixture contained 2.5 mL of 50 mmol/L phosphate buffer (pH 7.0), 0.4 mL of hydrogen peroxide, and 0.1 mL of enzyme extract. The decrease in absorbance at 240 nm was monitored to calculate CAT activity, which was expressed as U/g FW.

Peroxidase (POD) activity was determined using the guaiacol colorimetric method [24]. The reaction solution contained 0.05 mol/L PBS (pH 6.0), guaiacol, and H_2_O_2_. The reaction was initiated by adding the enzyme extract, and the increase in absorbance at 470 nm was measured. POD activity was expressed as U/g FW.

Ascorbate peroxidase (APX) activity was measured by monitoring the H_2_O_2_ dependent oxidation of ascorbate oxidation at 290 nm [30]. The decrease in absorbance at 290 nm was recorded, and APX activity were expressed in units per gram of fresh weight (U/g FW).

### 2.7. Determination of PAL Activity Together with Anthocyanin, Total Phenolics and Flavonoids Content

Phenylalanine ammonia-lyase (PAL) activity was determined according to the method described in [31]. A reaction mixture containing 2.9 mL of 0.02 moL/L L-phenylalanine and 0.1 mL of enzyme extract was incubated at 30 °C for 30 min. The reaction was terminated by rapid cooling on ice. For the blank, phosphate buffer was used instead of an enzyme extract. Absorbance was determined spectrophotometrically at 290 nm. One unit (U) of PAL activity was defined as the amount of enzyme causing a change of 0.01 in absorbance at 290 nm per hour under the assay conditions. PAL activity was calculated from the difference in absorbance between the sample and blank, taking into account the total enzyme extract volume, the volume of enzyme extract used in the reaction, the reaction time, and the fresh weight of the sample. PAL activity was expressed as U/g FW.

Total anthocyanins content (TAC), and total phenols (TPC) were determined using acidified methanol extracts as following method, 1.0 g of strawberry fruit was fully ground to homogenization with pre-cooled 1% HCl-methanol solution and fixed to 10 mL. After thorough mixing, the extraction was carried out at 4 °C, protected from light, for 20 min, with shaking during the period, and then centrifuged for 20 min at 4 °C, 4500 r/min. The TAC was calculated by measuring the absorbance values at 530 nm and 600 nm using a spectrophotometer. The value at 280 nm was expressed as the content of TPC. Favonoids content (FC) was determined using sodium nitrite-aluminum nitrate-sodium hydroxide colorimetric method at 510 nm [32]. Absorbance was measured at 510 nm. Flavonoid content was calculated using a rutin standard curve and expressed as mg/g FW.

### 2.8. RNA Sequencing and Metabolomics Analysis

To explore the mechanism of the inhibitory effect of MT+Se (designated as MS) treatment on gray mold incidence in strawberry fruits, qRT-PCR was applied to study gene expression at 0 h, 12 h, 24 h, 48 h, 72 h and 96 h. The results showed that the gene expression changes were highest at 72 h, when the strawberry fruits were just exhibiting the onset of the disease. Fruit samples at 0 and 72 h were thus selected for transcriptome analysis. Total RNA was extracted from strawberry fruit tissues using the Total RNA Extraction Kit for Plant Polysaccharides and Polyphenols (Tiangen, Beijing, China) according to the manufacturer’s instructions. Samples were collected at 0 h (CK-0, MS-0) and 72 h (CK-3, MS-3). RNA concentration and purity were assessed using a NanoDrop 2000 spectrophotometer (Thermo Scientific, Waltham, MA, USA).

mRNA was isolated using poly-T oligo-attached magnetic beads and fragmented using divalent cations. First-strand cDNA synthesis was performed with random oligonucleotides and SuperScript II, followed by second-strand synthesis with DNA Polymerase I and RNase H. Fragments were purified, adenylated at their 3′ ends, and ligated with Illumina PE adapters. Libraries were amplified via PCR and quantified using a Bioanalyzer 2100 system (Agilent, Santa Clara, CA, USA). Sequencing was performed on the NovaSeq 6000 platform (Illumina, San Diego, CA, USA). Genes with |log_2_Fold Change| > 1 and *p* < 0.05 were identified as differentially expressed genes (DEGs). Gene Ontology (GO) enrichment analysis was performed using topGO (v2.50.0), and Kyoto Encyclopedia of Genes and Genomes (KEGG) pathway enrichment analysis was performed using clusterProfiler (v4.6.0) software.

Eight genes were randomly selected for qRT-PCR analysis. Primers were designed using Primer Premier 5.0 (Table S5). Relative gene expression was calculated using the 2^−ΔΔCt^ method with three biological replicates [33]. A detailed description is provided in Section S2 of the Supplementary Materials.

Samples were the same as those used for RNA sequencing and were collected at 0 h (CK-0, MS-0) and 72 h (CK-3, MS-3). A total of 100 mg of strawberry fruit sample was placed in a 2 mL centrifuge tube; 500 µL of pre-cooled methanol:acetonitrile:water (2:2:1, *v*/*v*/*v*, containing 5 mg/kg 2-chlorophenylalanine) was added, followed by 2 steel beads, and the sample was vortex for 30 s. The samples were then placed in a high-throughput tissue grinder (TAITEC Corporation, Koshigaya, Saitama, Japan) and ground them for 60 s at 55 Hz, followed by sonication in an ultrasonic cleaner (KQ-500DE, Kunshan Ultrasonic Instruments Co., Kunshan, China) for 10 min after repeating once. After that, the samples were sonicated for another 10 min and then stored at −20 °C for 30 min, followed by centrifugation at 12,000 rpm for 10 min at 4 °C. The supernatant was passed through a 0.22 µm filter membrane, and the filtrate was transferred to sample vials for subsequent analysis.

DDA mass spectrometry data acquisition was performed using an ACQUITY UPLC HSS T3 column (Waters Corporation, Milford, MA, USA) and a Thermo Orbitrap Exploris 120 mass spectrometer (Thermo Fisher Scientific, Bremen, Germany) under the control of the software Xcalibur (version: 4.7, Thermo) for positive and negative ion modes. Metabolite identification was based on self-built libraries, mzCloud online library (https://www.mzcloud.org/), LIPID MAPS (https://www.lipidmaps.org/), HMDB (https://hmdb.ca/), MoNA (https://mona.fiehnlab.ucdavis.edu/), and the NIST-2020-MSMS mapping library.

### 2.9. Statistical Analysis

All experiments were conducted with three independent biological replicates (*n* = 3). Data are presented as mean ± standard deviation (SD). Statistical significance was determined using one-way analysis of variance (ANOVA), followed by Tukey’s multiple range test. Differences were considered statistically significant at *p* < 0.05. Data visualization and statistical analyses were performed using GraphPad Prism version 9.0 (GraphPad Software, San Diego, CA, USA) and SPSS Statistics version 25.0 (IBM Corp., Armonk, NY, USA).

## 3. Results

### 3.1. Inhibitory Effect of MT and Se on B. cinerea Mycelial Growth

**Figure 1:**
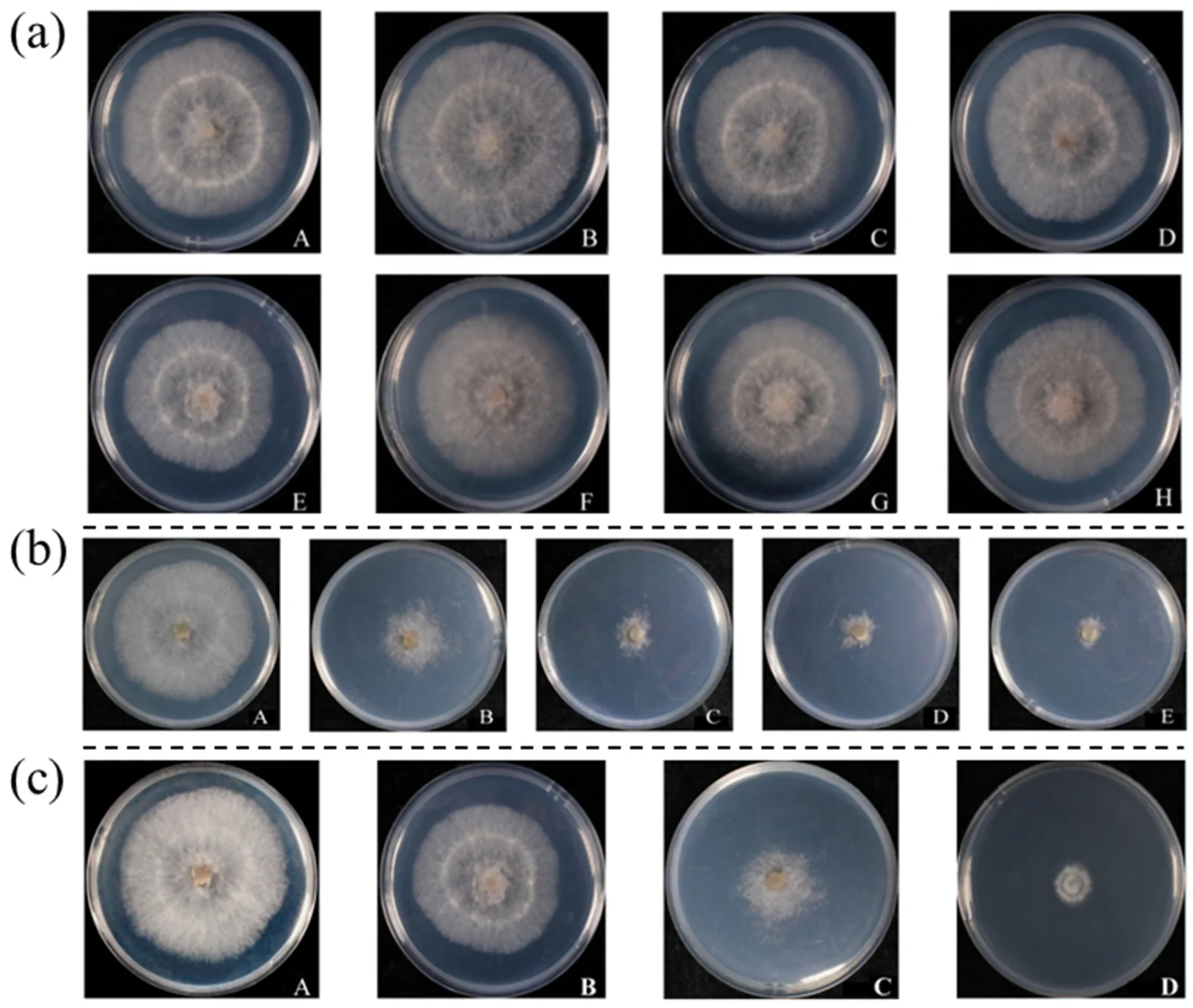
Effects of melatonin treatment (**a**), sodium selenite treatment (**b**), and combined MT+Se treatment (**c**) on the mycelial growth of *B. cinerea*. (**a**) A, sterile water control; B, 0.01 mmol/L MT treatment; C, 0.05 mmol/L MT treatment; D, 0.1 mmol/L MT treatment; E, 0.5 mmol/L MT treatment; F, 1 mmol/L MT treatment; G, 1.5 mmol/L MT treatment; H, 2 mmol/L MT treatment. (**b**) A, sterile water control; B, 20 mg/L Se treatment; C, 30 mg/L Se treatment; D, 40 mg/L Se treatment; E, 50 mg/L Se treatment. (**c**); A, control (CK); B, 0.5 mmol/L MT treatment; C, 25 mg/L Se treatment; D, combined treatment with 0.5 mmol/L MT and 25 mg/L Se.

Figure 1a illustrates the effect of melatonin (MT) on the mycelial growth of *B. cinerea*. As shown in Table S1, by day 3 of growth, *B. cinerea* exposed to various concentrations of MT exhibited no significant inhibition of mycelial extension. The impact of different concentrations of sodium selenite (Se) on *B. cinerea* mycelial growth is presented in Figure 1b. According to Table S2, the inhibitory effect on mycelial growth increased with rising Se concentrations. At 50 mg/L, sodium selenite achieved an inhibition rate of 87.74%, with an EC_50_ value calculated at 25 mg/L and a correlation coefficient of 0.98.

Based on these results and considering the potential synergistic effect of MT, a combined treatment of 0.5 mmol/L MT and 25 mg/L Se was selected. Sterile water served as the control (CK), MT alone was applied at 0.05 mmol/L, Se alone at 25 mg/L, and MT+Se represented the combination of 0.05 mmol/L MT and 25 mg/L Se. The effects of these treatments on *B. cinerea* mycelial growth are shown in Figure 1c. This indicates that the inhibitory rates of MT or Se applied individually were substantially lower than that of the combined MT+Se treatment. Notably, the MT+Se combination achieved a maximal inhibition rate of 93.67% (Table S3). Consequently, the composite formulation of 0.5 mmol/L MT and 25 mg/L Se was employed in subsequent experiments to further investigate its antifungal efficacy against *B. cinerea*.

### 3.2. Effects of MT+Se on the Morphology, Membrane Integrity, and Chitin Content of B. cinerea Hyphae

**Figure 2:**
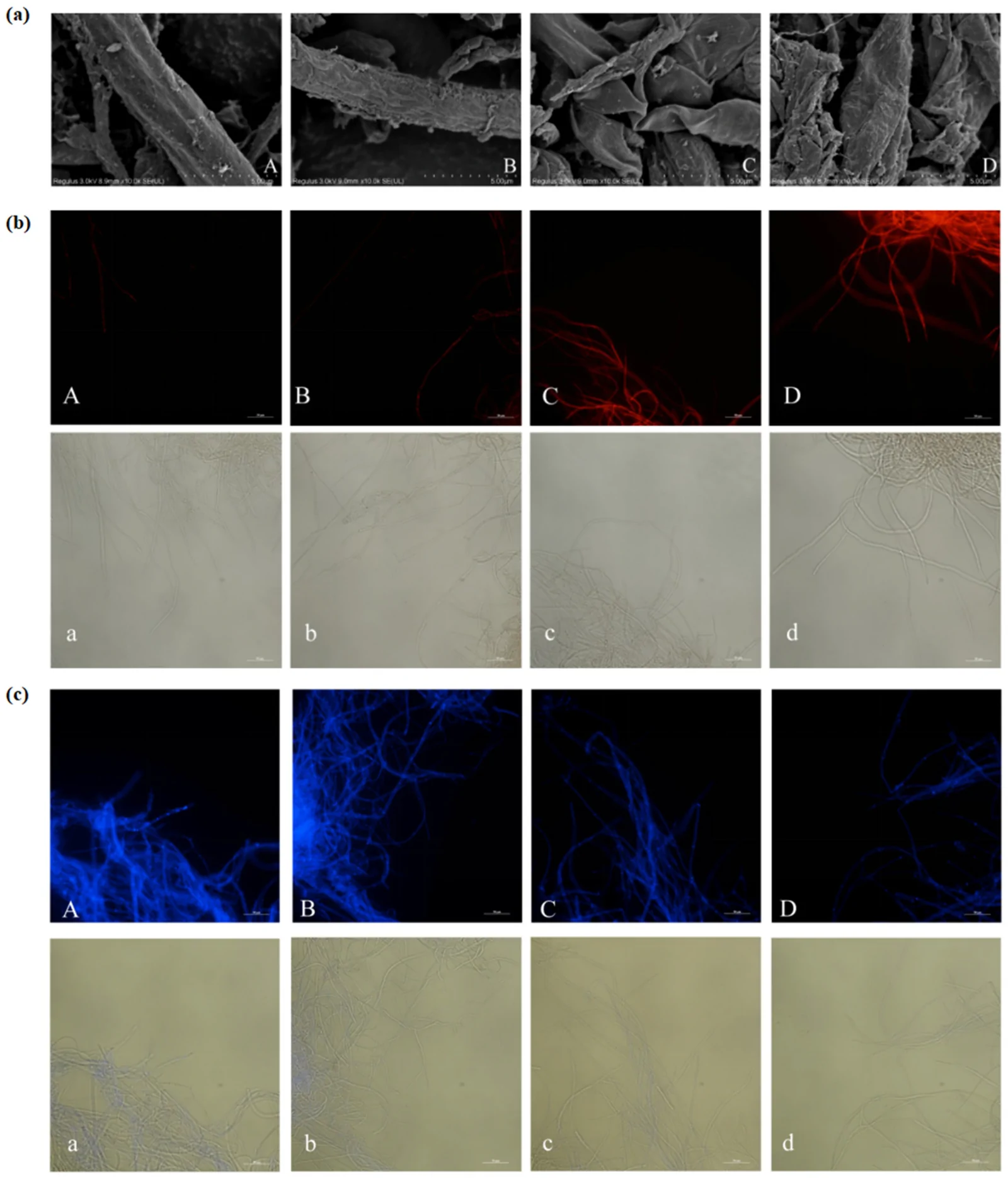
(**a**) SEM images showing the effects of MT, Se, and MT+Se treatments on the surface structure of *B. cinerea* hyphae. A: CK; B: 0.5 mmol/L MT; C: 25 mg/L Na_2_SeO_3_; D: 0.5 mmol/L MT + 25 mg/L Na_2_SeO_3_. (**b**) Propidium iodide (PI) staining to assess hyphal membrane integrity under different treatments. A/a: CK; B/b: 0.5 mmol/L MT; C/c: 25 mg/L Na_2_SeO_3_; D/d: 0.5 mmol/L MT + 25 mg/L Na_2_SeO_3_. Uppercase: bright-field; lowercase: red fluorescence. (**c**) Calcofluor White staining of hyphal chitin under different treatments. A/a: CK; B/b: 0.5 mmol/L MT; C/c: 25 mg/L Na_2_SeO_3_; D/d: 0.5 mmol/L MT + 25 mg/L Na_2_SeO_3_. Uppercase: bright-field; lowercase: blue fluorescence.

Scanning electron microscopy (SEM) and fluorescence staining analyses revealed that MT and Se, individually and in combination, differentially impacted the morphology and structural integrity of *B. cinerea* hyphae. SEM observations (Figure 2a) showed that in the CK control group, hyphae were plump and uniformly distributed, whereas 0.5 mmol/L MT treatment caused only minor surface wrinkling. In contrast, 25 mg/L Na_2_SeO_3_ treatment induced pronounced hyphal shrinkage and aggregation. The combined treatment of 0.5 mmol/L MT + 25 mg/L Na_2_SeO_3_ caused severe surface roughness, marked wrinkling, and extensive hyphal aggregation, indicating substantial disruption of hyphal surface architecture. Propidium iodide (PI) staining further demonstrated that MT+Se treatment compromised membrane integrity: CK hyphae exhibited negligible red fluorescence, 0.5 mmol/L MT caused weak fluorescence, 25 mg/L Na_2_SeO_3_ induced strong fluorescence, and the MT+Se combination markedly enhanced red fluorescence, indicating extensive membrane damage (Figure 2b). Calcofluor White staining revealed corresponding effects on cell wall chitin content: CK and MT-treated hyphae showed strong blue fluorescence, indicative of intact cell walls, whereas Se treatment reduced fluorescence, and the MT+Se combination almost completely abolished it, reflecting severe chitin depletion and cell wall disruption (Figure 2c). Collectively, these results suggest that MT+Se synergistically impairs hyphal structure, membrane integrity, and chitin accumulation, providing a potential mechanistic basis for its inhibitory effect on *B. cinerea* growth.

### 3.3. Effect of MT+Se Treatment on the Incidence of Gray Mold of Strawberry and the Essential Quality of Strawberry

**Figure 3:**
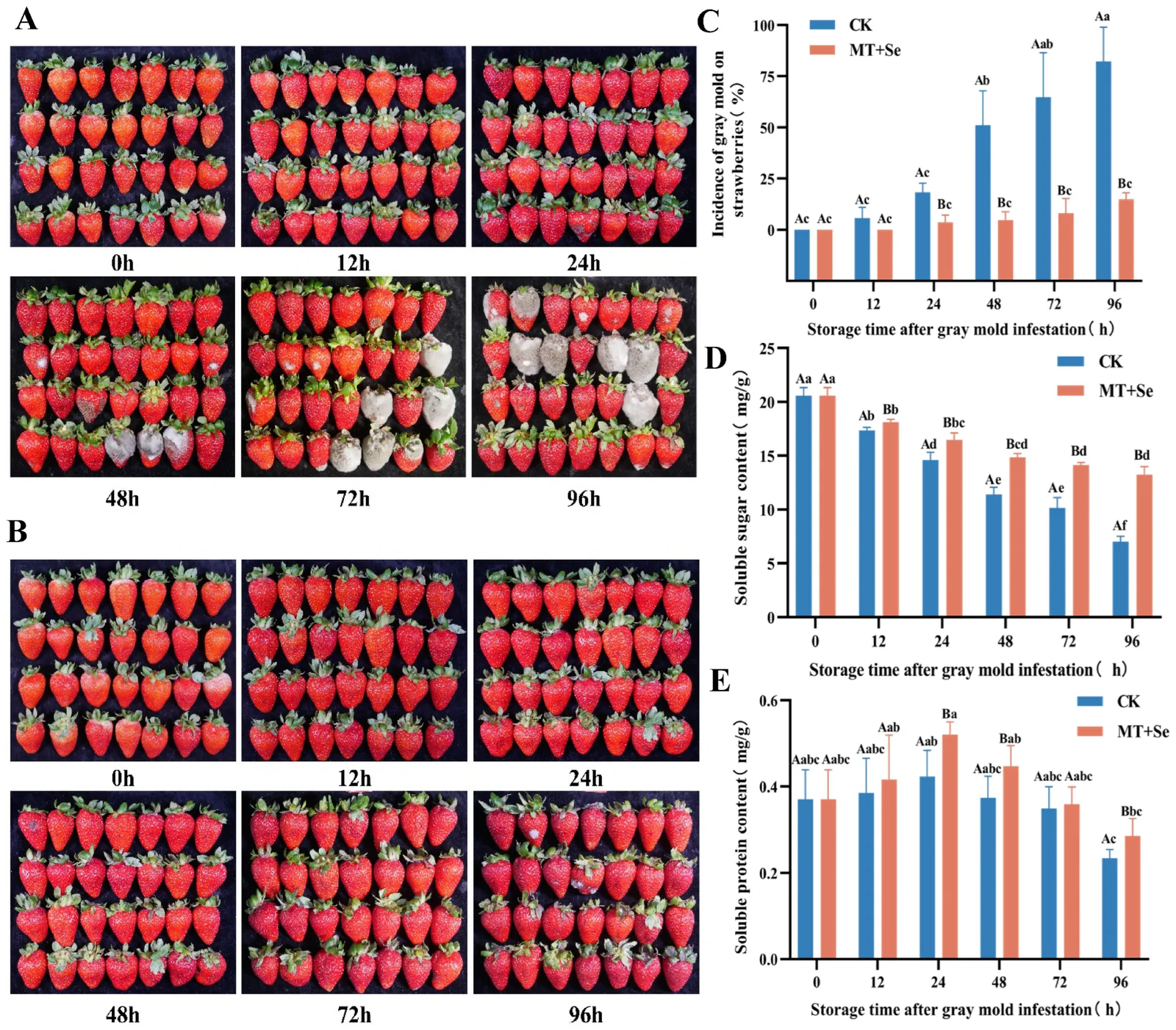
Effects of MT+Se treatment on strawberry fruit gray mold. (**A**) Strawberry fruits in the sterile water group (CK) at different sampling times. (**B**) Strawberry fruits treated with MT+Se at different sampling times. (**C**) Gray mold incidence in strawberry fruits. (**D**) Soluble sugar content in strawberry fruits. (**E**) Soluble protein content in strawberry fruits. Separate sets of fruits were independently evaluated at each time point, with 28 fruits per biological replicate and three independent biological replicates (*n* = 3). Data are presented as the mean ± SD. Different lowercase letters indicate significant differences among time points within the same treatment group; uppercase letters indicate significant differences between treatment groups at the same time point (*p* < 0.05).

The combined application of MT and sodium selenite (MT+Se) reduced the incidence of gray mold in strawberry fruits inoculated with *B. cinerea*. Gray mold incidence increased in the control group treated with sterile water (*p* < 0.05), reaching approximately 50% at 48 h and exceeding 75% by 96 h (Figure 3A,C). In contrast, strawberries treated with MT+Se exhibited lower gray mold incidence at 48, 72, and 96 h (*p* < 0.05), remaining below 15% throughout the observation period (Figure 3B,C). The soluble sugar content in strawberries declined over time. In the CK group, the soluble sugar content in strawberry fruits decreased from 20.59 mg/g to 7.01 mg/g. However, MT+Se slowed down this decrease (*p* < 0.05), and the soluble sugar content was still higher at 48 h, 72 h and 96 h compared to untreated fruits, and remained at 13.25 mg/g at 96 h (Figure 3D). Similarly, the soluble protein content of strawberry fruits initially increased, peaking at 0.520 mg/g at 24 h, and then gradually decreased with storage time, still containing 0.286 mg/L at 96 h. The MT+Se treatment enhanced the overall soluble protein content (*p* < 0.05) compared to the control group (Figure 3E).

### 3.4. Effect of MT+Se Treatment on Antioxidant Enzyme Activity and Content of Defense-Related Compounds

**Figure 4:**
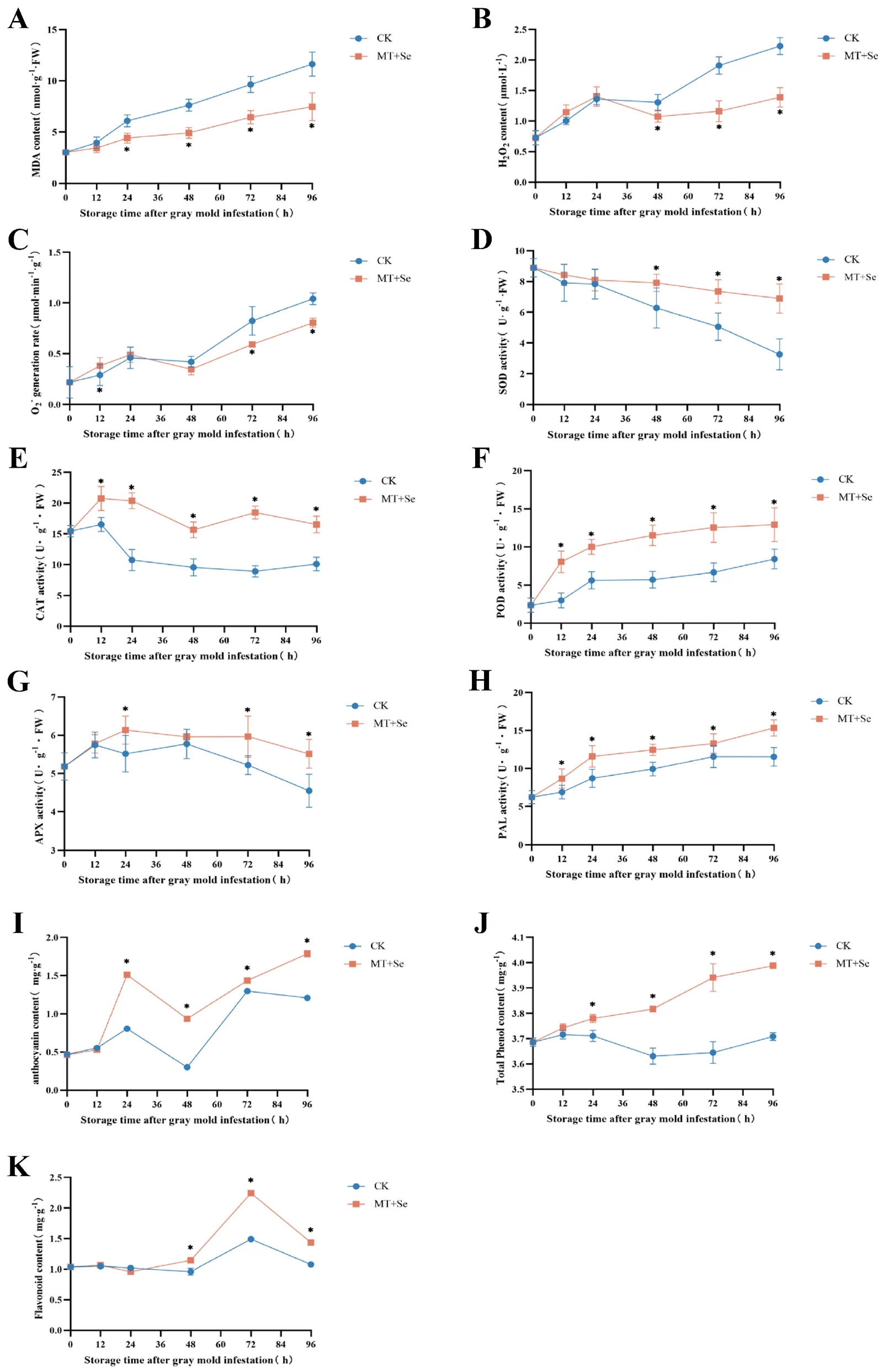
Effect of MT+Se treatment on oxidative stress indicators, antioxidant system, defense-related enzyme activities, and secondary metabolite contents in strawberry fruits during storage. (**A**) MDA content; (**B**) H_2_O_2_ content; (**C**) O_2−_ production rate; (**D**), SOD activity; (**E**) CAT activity; (**F**) POD activity; (**G**) APX activity; (**H**) PAL activity; (**I**) anthocyanin content; (**J**) total phenolic content; and (**K**) flavonoid content. Data are presented as mean ± SD of three independent biological replicates (*n* = 3). * Indicates a significant difference (*p* < 0.05) between the MT+Se group and the CK group at the same sampling time.

During the period of resistance to gray mold, the MDA content in strawberry fruits of CK group increased continuously from 3.03 nmol/g FW to 11.63 nmol/g FW, whereas in MT+Se-treated group, the MDA content reached 7.47 nmol/g FW at 96 h (Figure 4A). In the CK group, the H_2_O_2_ content increased from 0.727 µmol/L to 2.228 µmol/L, while in the MT+Se-treated group, H_2_O_2_ content increased to 1.402 µmol/L at 24 h, declined to 1.072 µmol/L at 48 h, and then gradually increased thereafter (Figure 4B). The O_2−_ production rate showed a similar temporal pattern. It increased continuously from 0.217 µmol/min/g FW to 1.04 µmol/min/g FW in the CK group, while in the MT+Se-treated group, it increased to 0.489 µmol/min/g FW at 24 h, decreased at 48 h, and then increased again from 48 to 96 h (Figure 4C).

In the CK group, SOD activity gradually decreased to 3.265 U/g FW, whereas SOD activity in the MT+Se-treated group was maintained at a higher level during the later storage period, reaching 6.89 U/g FW at 96 h (Figure 4D). Meanwhile, CAT activity showed different temporal patterns between the two groups. In the CK group, CAT activity increased initially and then decreased markedly, followed by a slight increase at 96 h. In contrast, the MT+Se-treated group maintained generally higher CAT activity from 12 to 96 h (Figure 4E). POD activity showed a continuous increase to 8.411 U/g FW at 96 h in the CK group, while the MT+Se-treated group showed consistently higher POD activity after treatment, reaching 12.918 U/g FW at 96 h (Figure 4F). APX activity fluctuated over time in both groups. The differences between the CK and MT+Se-treated groups varied among sampling times, and APX activity in the CK group was higher than that in the MT+Se-treated group at some intermediate time points, whereas MT+Se-treated fruits showed higher APX activity at 24, 72, and 96 h (Figure 4G).

PAL activity increased progressively during storage in both groups and was generally higher in MT+Se-treated fruits than in the CK group, with significant differences observed at multiple sampling times (Figure 4H). TAC showed marked fluctuations but was higher in MT+Se-treated fruits during the later storage period (Figure 4I). TPC content generally increased in MT+Se-treated fruits and remained higher than that in the CK group during the later storage period (Figure 4J). FC showed relatively small changes during the early storage period but increased markedly in MT+Se-treated fruits at 48–96 h, with the highest level observed at 72 h (Figure 4K). Overall, MT+Se treatment modulated antioxidant enzyme activities and defense-related biochemical responses in an enzyme- and time-dependent manner.

### 3.5. Transcriptome Data Quality Control Results, DEG, GO and KEGG Analysis

The sequencing quality control results (Table S6) revealed that Raw Q20 values exceeding 97% and Raw Q30 values above 94%. Reads with average quality scores below Q20 were filtered out during preprocessing to ensure Clean Reads quality. The Total Mapped Reads percentage was 82%, with 97% uniquely mapped to the reference genome. These metrics confirm that the transcriptome sequencing of all 12 samples was of high quality, providing reliable data for downstream analysis. And a total of 2952 differentially expressed genes (DEGs) were identified across the four experimental groups: CK 0 day, CK 3 day, MS 0 day, and MS 3 day, as illustrated in Figure S1.

GO enrichment analysis revealed that within the biological processes, enrichment was observed in pathways related to stimulus-response, hormone level regulation, and secondary metabolic processes. For cellular components, changes predominantly occur in the cell membrane. Molecular functions were primarily associated with oxidoreductase activity, with transcriptional regulator activity enriched explicitly in the MS-0 vs. MS-3 group (Figure S2). Regarding the KEGG-enriched pathways across the comparison groups, we found that the phenylpropanoid biosynthesis (fve00940) and flavonoid biosynthesis (fve00941), as well as the α-linolenic acid metabolism (fve00592) pathways, were commonly enriched (Figure S3). GO enrichment analyses focused on oxidase activity, whereas in KEGG analyses, the flavonoid synthesis pathway was almost always involved between comparison groups. These findings suggest that combined MT+Se treatment in strawberry fruit may enhance oxidative capacity and activate flavonoid biosynthetic pathways, thereby improving strawberry resistance to pathogenic bacteria.

### 3.6. Changes in Gene Expression of Oxidative Systems and Defense Substance Synthesis Pathways in Response to MT+Se

**Figure 5:**
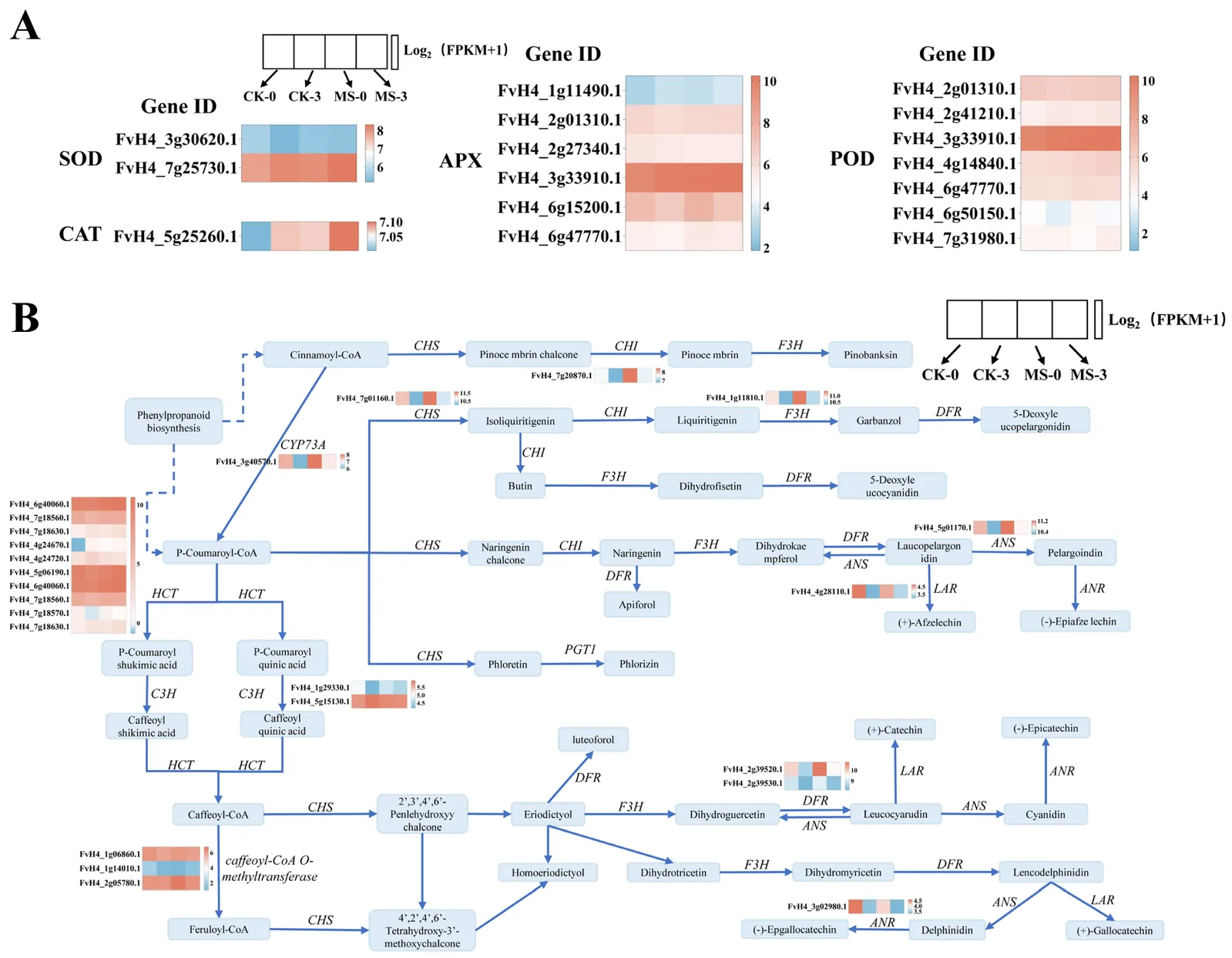
Transcription profiling of genes in MT+Se-treated strawberry fruits. Transcription profiling of genes related to antioxidant system (**A**) and flavonoid biosynthesis (**B**) in MT+Se-treated strawberry fruits.

Transcriptional profiling showed that postharvest treatment of strawberry fruit with MT+Se affected the expression of key genes for antioxidant enzymes and flavonoid synthesis. Comparing the antioxidant enzyme genes in the four groups of treatments revealed that the expression of SOD, CAT, POD, and APX genes was increased in the MT+Se treatment group of postharvest strawberry fruits, indicating that the MT+Se treatment improved the antioxidant capacity of postharvest strawberry fruits (Figure 5A).

The MT+Se treatment upregulated the expression of *trans-cinnamate 4-monooxygenase (CYP73A)*, *shikimate-O-hydroxycinnamoyltransferase (HCT)*, and *5-O-(4-coumaroyl)-D-quinate-3’-monooxygenase (C3’H)*, promoting the synthesis of caffeoyl-CoA (Figure 5B). Additionally, the upregulation of *caffeoyl-CoA-O-methyltransferase (CCoAOMT)* enhanced the production of feruloyl-CoA, further driving the synthesis of homoeriodictyol. Genes such as *CHS*, *CHI*, *naringenin-3-dioxygenase (F3H)*, *dihydroflavonol-4-reductase (DFR)*, and *anthocyanidin synthase (ANS)* were also upregulated, promoting the biosynthesis of phenolics like 5-deoxyleucopelargonidin, fisetinidol-4β-ol, pelargonidin, apiforol, and phloretin. However, the expression of *Leucoanthocyanidin reductase (LAR)* and *Anthocyanidin reductase (ANR)* was downregulated, limiting the reduction in phenolics such as leucocyanidin, leucodelphinidin, cyanidin, and delphinidin. These findings indicate that MT+Se treatment stimulates phenolic synthesis, while enhancing the antioxidant capacity of strawberry fruits.

### 3.7. Up-Regulated Resistance Genes and Key TFs in Response to MT+Se

To inquire further into the potential targets of disease resistance-related genes in strawberries enhanced by MT+Se treatment, according to the Plant Resistance Gene Database (PRGdb; http://prgdb.org/prgdb4/plants (accessed on 10 September 2025)), we targeted the CK-3 and MS-3 treatment groups. The Plant Resistance Gene Database identified ten upregulated DEGs associated with plant disease resistance in the CK-3 vs. MS-3 group. These genes belong to six structural domains listed in Supplementary Table S7. The upregulated genes include serine/threonine-protein kinase, receptor-like protein kinase, receptor-like serine/threonine-protein kinase, inactive protein kinase (associated with kinesin KIN), probable LRR receptor-like serine/threonine-protein kinase (part of receptor kinase RLK), and ABC transporter I family member 11 (belonging to nucleotide-binding domain N). Additionally, genes encoding a putative disease resistance protein (Nucleotide-binding domain and Leucine-rich repeat-containing receptor CNL), TMV resistance protein (linked to Toll-interleukin-1 receptor nucleotide-binding leucine-rich repeat TNL), and protein kinase PINOID (involved in Transket TRAN) were all upregulated. In addition to resistance-related genes, 35 upregulated transcription factors were identified in the CK-3 vs. MS-3 group, spanning 17 transcription factor families (Table 1).

These include ERF, HB-other, NF-YB, MYB, ARF, BES1, bHLH, MIKC-MADS, FAR1, C2H2, LBD, bZIP, MYB-related, NAC, HD-ZIP, SBP, and TALE. Within the ERF family, members such as *LURP-one-related 8*, *TRANSPARENT TESTA 12-like*, *ethylene-responsive transcription factor ERF061*, *cinnamoyl-CoA reductase 1-like*, and *LOC101295619* (an uncharacterised protein) were upregulated. The MYB family included *MYB-related protein 305-like*, *acid phosphatase 1*, and *MYB-related protein 306*, while the NAC family included *probable E3 ubiquitin-protein ligase ARI8*. These results suggest that MT+Se treatment induces the expression of key disease-resistance genes in strawberry fruits, enhancing their ability to combat pathogenic bacteria, and highlight that MT+Se treatment triggers the activation of multiple transcription factors involved in strawberry fruit resistance to pathogens.

**Figure 6:**
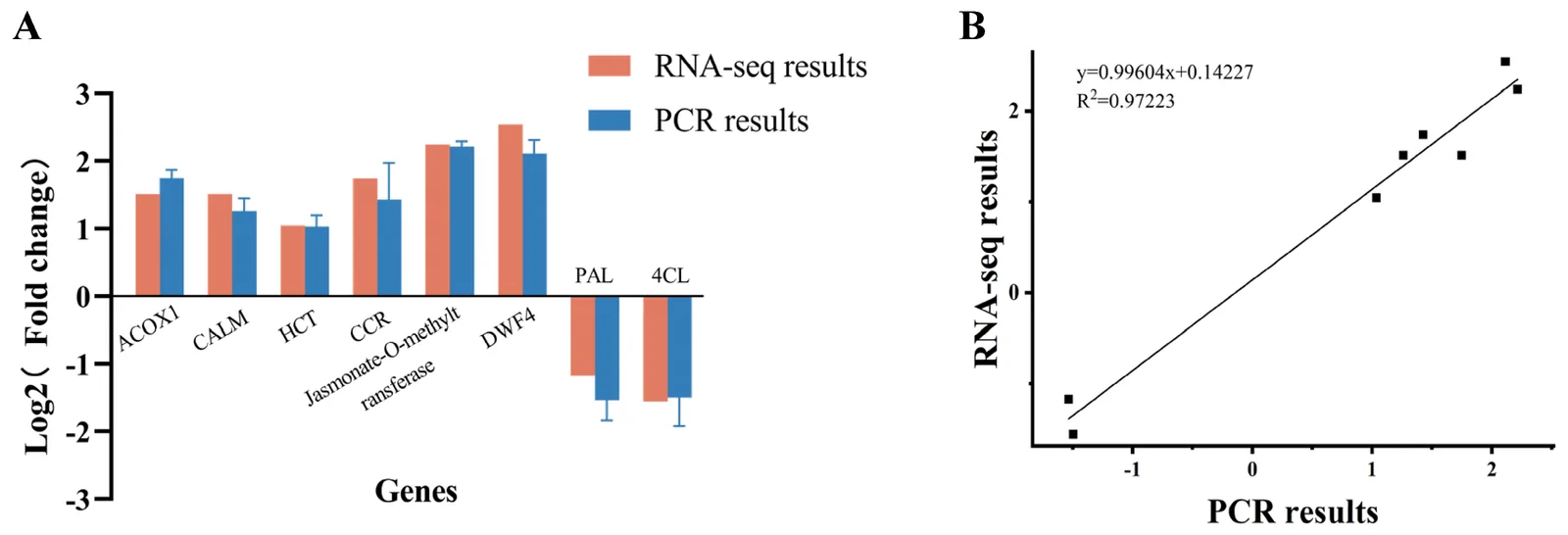
Transcriptome and qRT-PCR analysis of DEGs. Note: (**A**) DEGs transcriptome validation; (**B**) fit analysis.

To validate transcriptome data, genes such as *PAL*, *4CL*, *ACOX1*, *CALM*, *HCT*, *CCR*, *Jasmonate-O-methyltransferase*, and cytochrome P450 90B1 (*DWF4*) were randomly selected for qRT-PCR analysis across treatment groups (Figure 6). The qRT-PCR results were consistent with the transcriptome expression profiles, confirming the accuracy and reliability of the transcriptome data.

### 3.8. Metabolomic Data Quality Control Results, DEM, and KEGG Analyses

The mass spectra of the QC samples showed that the response intensities and retention times of the peaks of all QC samples basically overlapped, indicating that the variation caused by instrumental errors was small during the whole experiment (Figure S4A). The correlation coefficients of the QC samples in the correlation analysis all reached 0.99, which was extremely correlated, indicating that the similarity of expression between the QC samples was high (Figure S4B). The PCA of the experimental samples showed that the differences within the strawberry fruit samples of each treatment group were small and the differences in strawberry fruit samples between the treatment groups were obvious; moreover, the differences in the aggregation of QC samples were small in the PCA analysis (Figure S4C). Therefore, the correlation analysis of QC samples and PCA of strawberry fruit samples together indicated that the metabolome data were valid and reliable and a total of 602 differential metabolites were screened for metabolites in strawberry fruit samples (Figure S5).

KEGG pathway enrichment analysis revealed that the amino acid biosynthesis (map01230), linoleic acid metabolism (map00591), and α-linolenic acid metabolism (map00591) pathways were enriched in the CK-0 vs. CK-3 groups. The MS-0 vs. MS-3 groups exhibited enrichment in metabolic pathways, including flavonoid biosynthesis (map00941), flavonoid and flavonol biosynthesis (map00944), and anthocyanin biosynthesis (map00942). The CK-0 vs. MS-0 comparison group showed enrichment in the α-linolenic acid metabolism (map00592), isoflavonoid biosynthesis (map00943), flavonoid biosynthesis (map00941), and secondary metabolite biosynthesis (map01110) pathways. The flavonoid biosynthesis (map00941) and α-linolenic acid metabolism (map00592) pathways were significantly enriched in the CK-3 vs. MS-3 comparison group (Figure S3).

### 3.9. Co-Enrichment Pathway Differential Metabolite Changes

**Figure 7:**
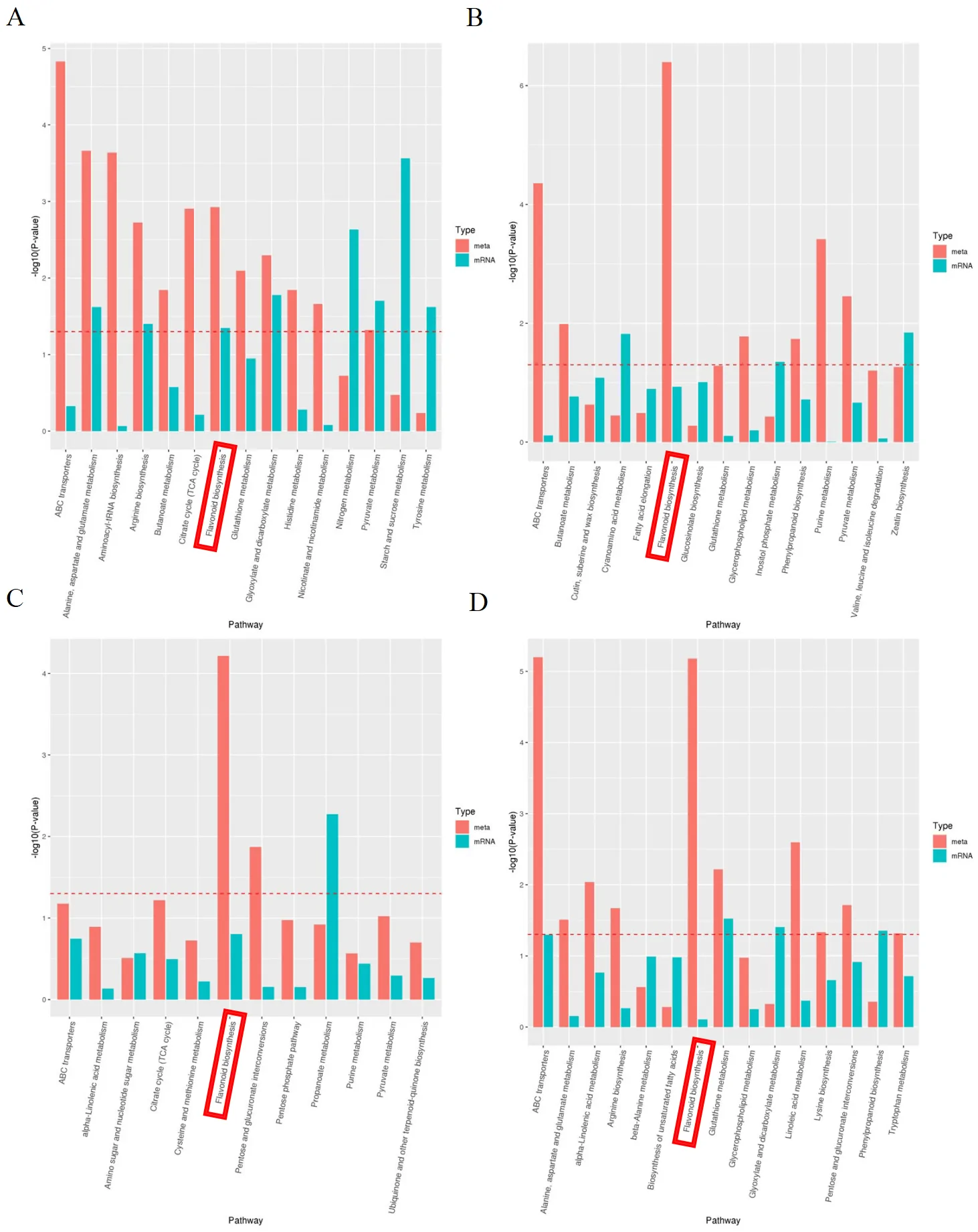
Transcriptome-metabolome co-enrichment pathway. KEGG pathway analysis of transcriptome and metabolome co-enriched DEGs (blue columns) and DEMs (red columns). (**A**) CK-0 vs. CK-3; (**B**) MS-0 vs. MS-3; (**C**) CK-0 vs. MS-0; (**D**) CK-3 vs. MS-3. The red dashed line indicates the significance threshold (*p* = 0.05), and the red boxes highlight flavonoid biosynthesis in panels (**A**–**D**).

Comparative analysis of transcriptomic and metabolomic pathways enriched in KEGG. Alanine, aspartate, and glutamate metabolism, along with flavonoid biosynthesis pathways, exhibited co-enrichment within the CK-0 vs. CK-3 group. Similarly, flavonoid biosynthetic pathways were co-enriched in the MS-0 vs. MS-3 group, also implicating phenylpropanoid biosynthesis. The CK-0 vs. MS-0 group demonstrated co-enrichment in flavonoid biosynthesis and α-linolenic acid metabolic pathways. Meanwhile, the CK-3 vs. MS-3 group displayed co-enrichment in flavonoid biosynthesis, phenylpropanoid biosynthesis, and the α-linolenic acid metabolic pathway. Notably, the flavonoid biosynthetic pathway was significantly enriched across all treatment groups (Figure 7).

**Figure 8:**
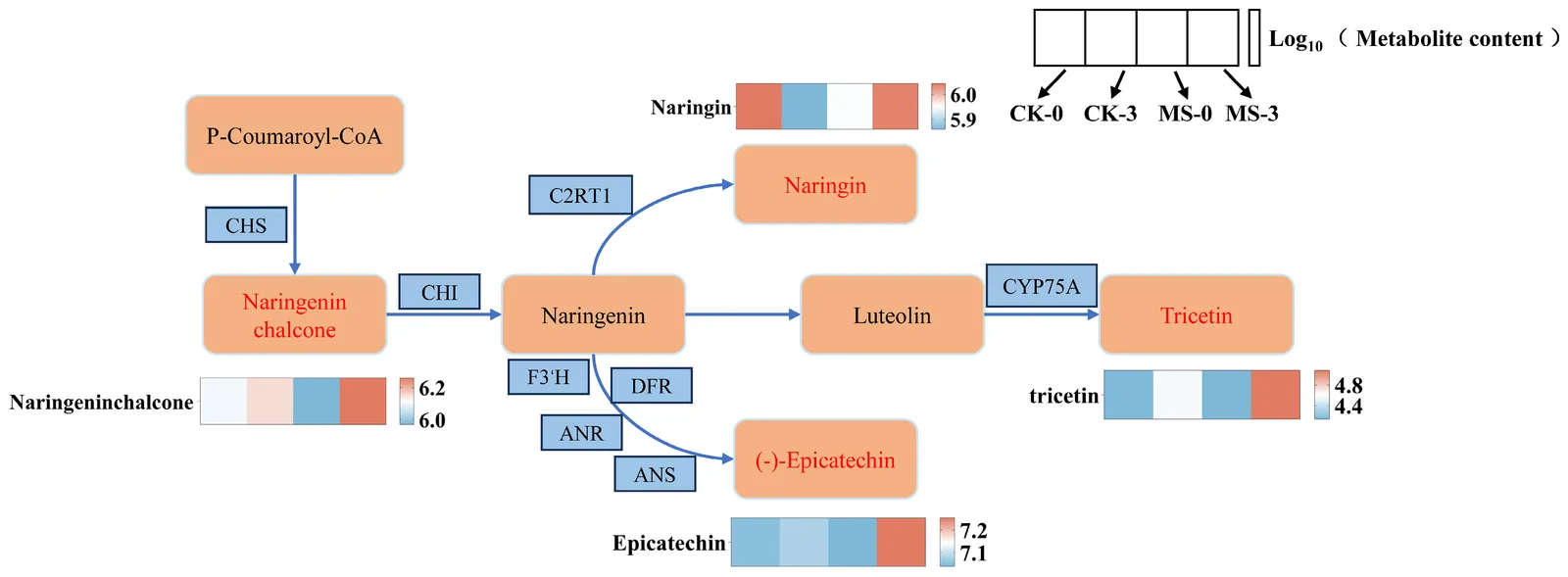
Expression profiles of some DEMs in the flavonoid pathway. Changes in the strawberry fruit flavonoid biosynthesis pathway after MT+Se treatment. Blue boxes indicate relevant genes involved in the pathway, orange boxes are relevant metabolites, and red text indicates that the accumulation of the differential metabolite increased after MT+Se treatment.

Among the co-enriched flavonoid biosynthetic pathways, the content of naringenin chalcone, naringenin, tricetin and epicatechin were at a higher level in the MS-3 treated group compared to the CK-3 group. In the co-enriched flavonoid biosynthesis pathway, naringenin chalcone, narigin, tricetin, and epicatechin contents were significantly higher in the MS-3 treatment group compared to the CK-3 group. Combined transcriptome gene expression showed that in the MS-3 treatment group, MT+Se treatment up-regulated the expression of *CHS* to increase the accumulation of naringenin chalcone content, while at the same time, the up-regulation of the expression of *CHI* indirectly contributed to the increase in the content of narigin, as well as tricetin; in addition, the up-regulation of the expression of *F3’H*, *DFR*, *ANR*, and *ANS* also led to an increase in epicatechin content (Figure 8). These findings suggest that MT+Se treatment can promote the synthesis of secondary metabolites by affecting the regulation of gene expression within the flavonoid pathway, leading to the enhancement of strawberry resistance to gray mold.

## 4. Discussion

In the fungal activity assay in vitro, contrary to the results of melatonin in improving resistance to gray mold in cherry tomatoes, MT did not significantly inhibit the growth of gray mold mycelia in vitro, suggesting that MT may contribute to disease resistance primarily through modulation of host defense responses rather than through a direct inhibitory effect on *B. cinerea* [34]. However, Similar to the inhibition of *S. sclerotiorum* mycelial growth by Se, our study confirmed that Se also inhibited the mycelial growth of gray mold, and the degree of inhibition was positively correlated with the concentration [35]. Further, the inhibition of gray mold mycelial growth could reach 93.67% after the combined application of 0.5 mmol/L MT and 25 mg/L Se. MT has been shown to inhibit the spore germination of a variety of fungi, as well as affect the mycelium [36]. The stronger inhibitory effect observed with the combined MT+Se treatment than with either treatment alone suggests that the two agents may interact in suppressing *B. cinerea* growth. Our study was similar to the growth inhibition of gray mold by cinnamaldehyde, but the combination of MT+Se broke the time limitation of cinnamaldehyde on the growth inhibition of gray mold and directly inhibited the growth of gray mold mycelium [37].

Gray mold is highly destructive to strawberries, affecting their growth and storage at all stages [38]. Both MT, a plant signaling molecule, and Se, a beneficial trace element, have been reported to contribute to plant stress responses [39]. This study treated postharvest strawberries with MT+Se and evaluated changes in antioxidant capacity, defense-related compounds, and the expression of disease resistance-related genes. These responses were associated with enhanced defense and maintenance of fruit quality. Physiological and biochemical analyses of control (CK) and treated (MT+Se) fruits and transcriptome profiling of strawberries inoculated with gray mold, revealed resistance genes and transcription factors associated with MT+Se treatment.

The results of the present study showed that MT+Se treatment greatly reduced the incidence of gray mold at the macroscopic level. At 96 h, gray mold incidence was substantially lower in MT+Se-treated fruits than in the CK group. Previous studies have also demonstrated the potential of exogenous treatments for controlling strawberry gray mold. For example, tropolone 1000 and 2000 mg/L significantly reduced gray mold incidence in strawberry fruits [40]. Similarly, in the present study, MT+Se treatment markedly suppressed gray mold development, with disease incidence remaining below 15% at 96 h compared with more than 75% in the CK group. The effect of MT+Se on the basic quality of strawberry fruits was also evaluated. Strawberry fruits treated with MT+Se maintained higher soluble sugar and soluble protein contents than the CK fruits during storage. In the CK group, the soluble sugar content decreased by about 65% and the soluble protein content decreased by about 27%, whereas the soluble sugar content decreased by about 35% and the soluble protein content decreased by about 20% in the MT+Se-treated group. Soluble sugars serve as crucial osmolytes in plants, helping maintain internal balance under stress conditions [41]. Studies have shown that plants under abiotic stress, such as drought, accumulate soluble sugars to mitigate damage [42]. Similarly, our findings indicate that MT+Se treatment delays the reduction in soluble sugar content in strawberries infected with gray mold, suggesting that biotic stress also influences sugar regulation. Soluble proteins, essential for osmoregulation, were also maintained at higher levels following MT+Se treatment, consistent with findings in grapevines treated with biodegradable liquid films to enhance cold resistance [43]. Together, these observations suggest that MT+Se treatment contributed to the maintenance of soluble sugar and protein contents during gray mold development.

The antioxidant enzyme system maintains plants’ reactive oxygen species (ROS) homeostasis, elevated ROS levels can damage plant cells, while insufficient ROS can hinder defensive responses [44]. Enzymes such as SOD, CAT, POD, and APX scavenge excess ROS and mitigate oxidative damage [45]. Our results showed that MT+Se treatment enhanced antioxidant enzyme activities in an enzyme- and time-dependent manner. In particular, MT+Se-treated fruits generally maintained higher SOD, CAT, and POD activities during storage, although the response patterns varied among individual enzymes and sampling times. Meanwhile, lower MDA accumulation and altered H_2_O_2_ and O_2_^−^ dynamics were observed in MT+Se-treated fruits compared with CK fruits. These findings are broadly consistent with previous studies showing that exogenous salicylic acid and MT can modulate antioxidant defense systems under biotic or abiotic stress [46,47]. Se application has also been reported to reduce the incidence or development of fungal diseases in different plant systems [47], supporting its potential role in plant disease resistance. Together, these findings suggest that MT+Se treatment may contribute to maintaining ROS homeostasis and alleviating oxidative damage in postharvest strawberry fruits through modulation of antioxidant defense responses.

Further evaluation of the effect of MT+Se treatment on secondary metabolite content of strawberry fruits revealed that MT+Se treatment altered the accumulation of anthocyanins, total phenolics, and flavonoids during storage, with generally higher levels observed in MT+Se-treated fruits at several sampling times. Anthocyanins, total phenolics, and flavonoids are key secondary metabolites with potent antioxidant and antimicrobial properties [48]. These compounds protect plants from oxidative damage and pathogens, while enhancing fruit ripeness and freshness [49]. Flavonoids, total phenolics and other antibacterial actives that the plant itself can produce are looked for in most plant disease studies, and the high polyphenol and flavonoid content adds antimicrobial power to the Koiwaiko lily leaf and flower extracts [50]. The application of MT+Se differs from similar exogenous increases in antimicrobial substances such as flavonoids and total phenolics. Instead, it induces the production of such chemicals by the plant itself, identical to the effect of 2, 3-Butanediol in mitigating brown spot of creeping bentgrass, and consistent with the application of *Trichoderma virens 6PS-2* to improve the consistency of *Fusarium* in *M. hupehensis* seedlings [51]. Thus, the changes observed in the present study suggest that modulation of secondary metabolite accumulation may contribute to the response of strawberry fruits to MT+Se treatment.

Transcriptome analysis showed that MT+Se treatment up-regulated the expression of antioxidant enzyme genes *SOD*, *POD*, *CAT*, and *APX*. Up-regulated expression of antioxidant enzyme-related genes may contribute to enhanced antioxidant capacity and reduce oxidative stress in plants [52]. The findings of our study were consistent with those of a previous study showing that exogenous salicylic acid treatment promoted the expression of antioxidant enzyme-related genes in *Panax vietnamensis* [53]. Together with the observed changes in antioxidant enzyme activities and ROS levels, these transcriptional responses suggest that antioxidant regulation may contribute to ROS homeostasis and the defense response of strawberry fruits against gray mold. Meanwhile, our results indicate that MT+Se treatment up-regulated flavonoid biosynthesis genes such as *CYP73A*, *HCT*, *C3’H*, *CHS*, *CHI*, *F3H*, *DFR*, and *ANS*, and induced the accumulation of flavonoid-related metabolites such as pelargonidin and fisetinidol, consistent with previous observations that post-harvest UV-C treatment modulated phenylpropanoid and flavonoid metabolism in sweet cherries [54]. Similarly, combined resveratrol and nitric oxide treatment has been reported to enhance resistance to gray mold in tomato through defense-related responses [55]. Taken together, our transcriptomic and metabolomic results suggest that the response of strawberry fruits to MT+Se treatment may involve coordinated regulation of antioxidant defenses and secondary metabolic pathways.

MT+Se treatment up-regulated 10 plant disease resistance-related genes, and several genes involved in protein serine/threonine kinases were significantly enriched, as was the case with exogenous salicylic acid (SA) for initiating the defense response of the tetraploid potato *SD20* and its application in multiple signaling pathways [56]. Among them, *serine/threonine-protein kinase* enhanced plant resistance to stresses [57]. The *putative disease resistance RPP13-like protein 3* is involved in abscisic acid-mediated heat stress response in maize. Thus, others have hypothesized that the *putative disease resistance protein RGA3 isoform X1* also possesses stress resistance capacity [58]. In research on soybean mosaic virus, gene annotation identified the three candidate genes, including one NBS-LRR type gene and two serine/threonine protein type genes, which may be related to the resistance to SC18 [59]. Therefore, based on these observations and previous studies, the probable LRR receptor-like serine/threonine-protein kinase At4g26540 identified in the present study may be associated with the defense response of strawberry fruits to MT+Se treatment. However, its specific role in resistance to *B. cinerea* remains to be experimentally validated. These data suggest that such genes are potential disease resistance-associated factors in response to MT+Se treatment, but more experimental evidence is needed to their specific functions.

Moreover, 35 upregulated transcription factor genes belonging to 17 families, including ERF, bHLH, NAC, and MYB, were identified as upregulated. These families regulate stress responses, secondary metabolism, and hormone signalling [60]. Among the differentially expressed genes, *cinnamoyl-CoA reductase 1-like* and *ethylene-responsive transcription factor ERF061* were upregulated following MT+Se treatment. Previous studies have implicated *CCR* was shown to enhance resistance to *Magnaporthe grisea* and *Xanthomonas oryzae pv. oryzae* (*Xoo*) in rice is hypothesized that *cinnamoyl-CoA reductase 1-like* may similarly have potential disease resistance properties [61]. Our results are consistent with the fact that hormone treatments such as exogenous SA and methyl jasmonate (MeJA) significantly induced the expression of maize *ZmERF061* to enhance resistance to *Exserohilum turcicum*, and that MT+Se treatment increased the expression of *ethylene-responsive transcription factor ERF061* that improved the resistance of strawberry to gray mold [62]. In the study of color regulation in Rhododendron latoucheae, others found that the up-regulated expression of bHLH130, a transcription factor in the bHLH family involved in flavonoid biosynthesis, affected the color of R. latoucheae, which could be a potential factor for inducing MT+Se treatment to maintain the freshness of strawberry fruits [63]. The MYB family of *acid phosphatase 1* can act as an active acid phosphatase (*OsACP1*) in rice to regulate rice growth under intracellular phosphate (Pi) stress conditions by recycling Pi in organic phosphate (Po) in ER and GA, which indirectly suggests that MT+ Se-treated transcription factor *acid phosphatase 1* can maintain the physiological, environmental balance of fruit cells during gray mold infestation and still effectively maintain fruit quality [64]. In plants, E3 ubiquitin ligases are the core of phytohormone regulation, including jasmonic acid, auxin and many other hormones. The expression of *probable E3 ubiquitin-protein ligase ARI8* was elevated, which may be a prerequisite for the response of the combined oxidative system and the pathways of various defense substances in strawberry fruits after MT+Se treatment. While their specific regulatory roles in strawberry defense require further investigation, these findings suggest their importance in MT+Se-mediated disease resistance and provide potential targets for resistance to gray mold of strawberry.

**Figure 9:**
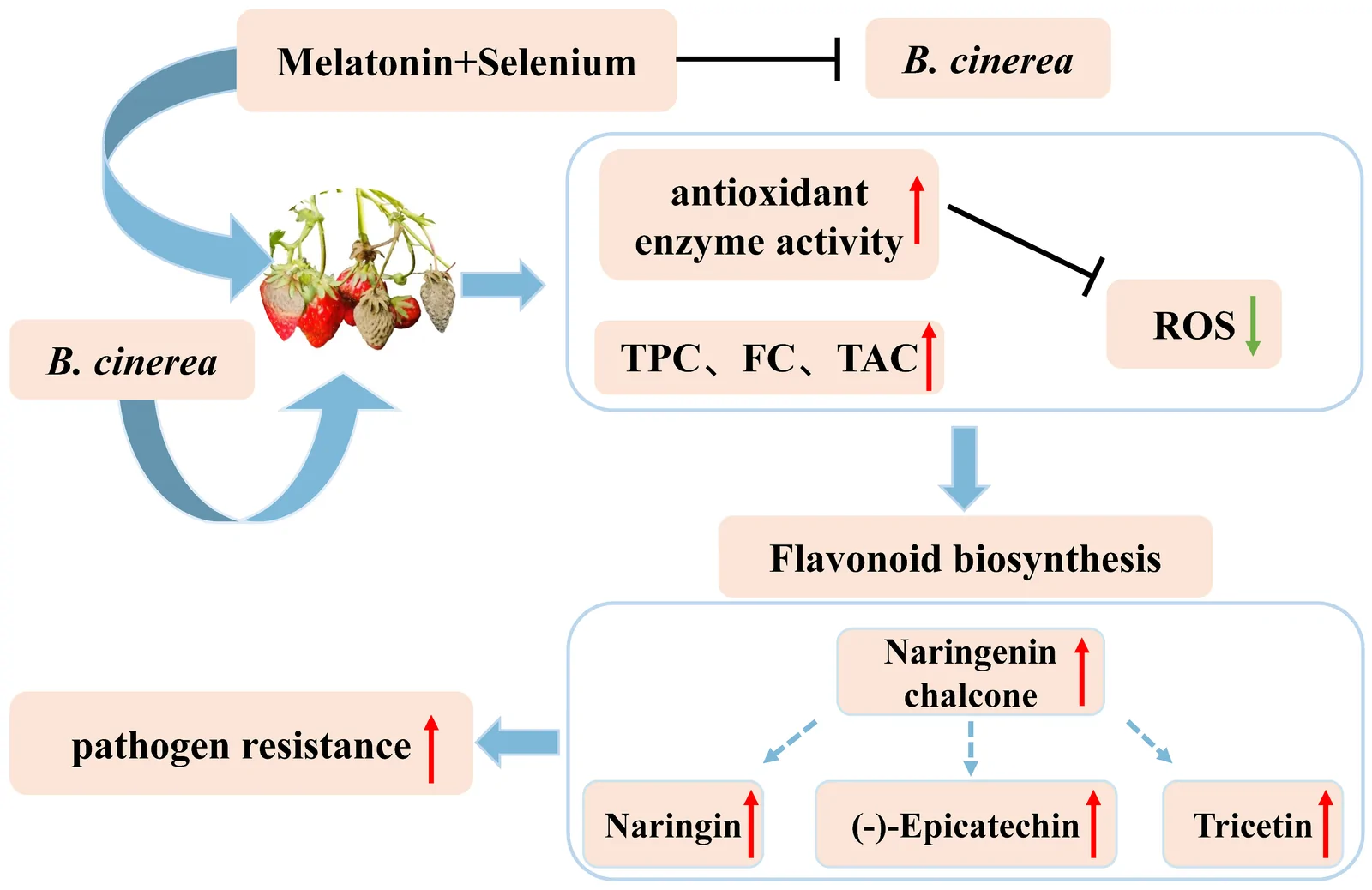
Possible mechanisms of postharvest MT+Se treatment in strawberries to reduce gray mold. The MT+Se combination treatment directly inhibited the growth of gray mold in vitro, induced the enhancement of the oxidative system in strawberry fruits, promoted the synthesis of secondary metabolites, and modulated the flavonoid biosynthesis pathway to increase the accumulation of antimicrobial substances, contributing to the suppression of gray mold in strawberry fruits. Red upward arrows and green downward arrows indicate increases and decreases, respectively; blue dashed arrows indicate the relationships within the flavonoid biosynthesis pathway.

Metabolomic analyses showed that, unlike thiamine, which modulates the phenylpropane pathway in muskmelon fruits in response to black spot disease, MT+Se treatment affected the accumulation of flavonoid metabolic pathways [65]. Naringenin chalcone, naringenin, tricetin, and epicatechin are all naturally occurring antimicrobial compounds found in plants. Naringin Chalcone possesses antimicrobial activity against phytopathogenic bacteria and improves the resistance of tobacco to *Phytophthora nicotianae*. Naringenin improves the antimicrobial activity of Arabidopsis thaliana against *Pseudomonas syringae* [66]. Exogenous epicatechin synergized with caffeic acid has been shown to inhibit gray mold infestation in apples [67]. Our experiments demonstrated that exogenous MT+Se treatment increased the accumulation of naringenin chalcone, naringenin, tricetin, and epicatechin. Combined with the transcriptomic results showing altered expression of genes involved in flavonoid biosynthesis, these findings suggest that MT+Se treatment may modulate flavonoid biosynthesis and thereby contribute to the observed resistance of strawberry fruits to gray mold. These results suggest that MT+Se treatment is associated with changes in the expression of genes related to the flavonoid pathway and the accumulation of antimicrobial substances, which may contribute to the disease resistance of strawberry fruits (Figure 9). Nevertheless, the fruit experiment included only CK and MT+Se treatments, which limits the assessment of the individual contributions and potential interactive effects of MT and sodium selenite in vivo. In addition, further studies under broader postharvest storage conditions are needed to evaluate the general applicability of the observed effects.

## 5. Conclusions

This study demonstrates that melatonin (MT) and sodium selenite treatment enhance postharvest resistance of strawberry fruits to *B. cinerea* and is associated with coordinated physiological, biochemical, transcriptomic, and metabolomic mechanisms. The combined MT+Se treatment exhibited a markedly stronger inhibitory effect on *B. cinerea* in vitro than either MT or sodium selenite alone, with an inhibition rate of 93.67%, and maintaining gray mold incidence below 15% in treated fruits. MT+Se preserved fruit quality by sustaining higher soluble sugar and protein contents while mitigating oxidative damage, as evidenced by lower MDA and H_2_O_2_ levels and enhanced antioxidant enzyme activities (SOD, CAT, POD, APX). Transcriptomic analyses revealed upregulation of antioxidant enzymes, resistance-related genes, and multiple transcription factors, whereas metabolomic profiling showed increased accumulation of defense-related secondary metabolites, particularly flavonoids and phenylpropanoids such as naringenin chalcone, narigin, tricetin, and epicatechin. Co-enrichment analysis of transcriptomic and metabolomic data indicated that response to MT+Se treatment was associated with changes in flavonoid and α-linolenic acid metabolic pathways, which may contribute to observed antioxidant defenses and disease resistance responses. Overall, these findings indicate that combined MT+Se treatment is associated with enhanced resistance of strawberry fruits to *B. cinerea*, accompanied by coordinated changes in antioxidant defense, secondary metabolism, and resistance-related molecular responses. These results provide a basis for further investigation of MT and sodium selenite as potential treatments for the postharvest management of gray mold in strawberry fruit.

## Figures and Tables

**Table 1:** Up-regulated differentially expressed TFs in response to MT+Se treatment.

Gene ID	TFs	log_2_FoldChange	Description
FvH4_6g09160.1	ERF	1.76896	PREDICTED: protein LURP-one-related 8
FvH4_6g01120.1	HB-other	2.337249	PREDICTED: serine/threonine-protein kinase At3g07070-like
FvH4_5g16670.1	NF-YB	1.52442	PREDICTED: L-Ala-D/L-amino acid epimerase
FvH4_7g30070.1	ERF	1.188578	PREDICTED: protein TRANSPARENT TESTA 12-like
FvH4_6g50930.1	MYB	1.226055	PREDICTED: myb-related protein 305-like
FvH4_7g19350.1	ARF	1.550859	PREDICTED: auxin response factor 18-like
FvH4_6g07990.1	BES1	2.025438	PREDICTED: nodulin-26-like
FvH4_5g04470.1	ERF	1.080733	PREDICTED: ethylene-responsive transcription factor ERF061
FvH4_7g25860.1	bHLH	1.369626	PREDICTED: expansin-A10
FvH4_6g37880.1	MIKC_MADS	1.009811	PREDICTED: floral homeotic protein AGAMOUS isoform X1
FvH4_6g28680.1	ERF	1.741186	PREDICTED: cinnamoyl-CoA reductase 1-like
FvH4_4g28630.1	FAR1	1.19612	PREDICTED: protein STRICTOSIDINE SYNTHASE-LIKE 10-like
FvH4_5g37290.1	C2H2	1.039184	uncharacterized protein LOC101311001
FvH4_3g07420.1	LBD	1.373368	PREDICTED: cellulose synthase A catalytic subunit 8
FvH4_2g10770.1	bHLH	5.440476	PREDICTED: auxin-induced protein 15A-like
FvH4_4g18540.1	ERF	1.778881	PREDICTED: uncharacterized protein LOC101295619
FvH4_2g04900.1	bHLH	1.248583	PREDICTED: transcription factor bHLH113-like isoform X2
FvH4_7g03430.1	bZIP	1.83792	PREDICTED: LOW-QUALITY PROTEIN: dynamin-related protein 4C-like
FvH4_3g38290.1	C2H2	1.058524	PREDICTED: uncharacterized protein LOC101298583
FvH4_6g08000.1	BES1	1.300999	PREDICTED: aquaporin NIP1-1-like
FvH4_3g41850.1	MYB_related	1.078889	PREDICTED: probable amino acid permease seven isoform X3
FvH4_1g22110.1	NF-YB	1.2916	PREDICTED: nuclear transcription factor Y subunit B-3-like
FvH4_3g24920.1	NAC	2.168364	PREDICTED: probable E3 ubiquitin-protein ligase ARI8
FvH4_3g08560.1	HB-other	1.304055	PREDICTED: triose phosphate/phosphate translocator TPT, chloroplastic isoform X1
FvH4_5g19570.1	HD-ZIP	1.404168	PREDICTED: homeobox-leucine zipper protein HAT4-like
FvH4_7g16110.1	bHLH	1.145998	PREDICTED: hydroquinone glucosyltransferase-like
FvH4_1g06500.1	MYB	1.173946	PREDICTED: acid phosphatase 1
FvH4_3g19410.1	NAC	1.019925	PREDICTED: protein CUP-SHAPED COTYLEDON 1-like
FvH4_6g20610.1	bZIP	1.202722	PREDICTED: basic leucine zipper 43-like
FvH4_5g03880.1	bZIP	1.09693	PREDICTED: common plant regulatory factor 1-like
FvH4_6g53710.1	SBP	1.102479	PREDICTED: uncharacterized protein LOC101306079
FvH4_6g14170.1	C2H2	1.294807	PREDICTED: short-chain dehydrogenase TIC 32, chloroplastic
FvH4_6g48630.1	MYB	1.030161	PREDICTED: myb-related protein 306
FvH4_6g38080.1	TALE	1.003727	PREDICTED: trifunctional UDP-glucose 4,6-dehydratase/UDP-4-keto-6-deoxy-D-glucose 3,5-epimerase/UDP-4-keto-L-rhamnose-reductase RHM1-like
FvH4_6g33080.1	MYB_related	1.173432	PREDICTED: inactive protein kinase SELMODRAFT_444075

*Note:* Gene ID: Strawberry fruit transcriptome sequencing gene numbering; TFs: Family of transcription factors in which the gene resides; log_2_FoldChange: Fold difference in gene expression between CK-3 and MS-3 treatment groups; Description: Gene description according to NCBI.

## Data Availability

The original contributions presented in this study are included in the article/Supplementary Materials. Further inquiries can be directed to the corresponding authors.

## Supplementary Materials

The following supporting information can be downloaded at https://www.mdpi.com/article/10.3390/foods15183291/s1: Table S1: Inhibition of mycelial growth of *B. cinerea* by melatonin treatment; Table S2: Inhibition of mycelial growth of *B. cinerea* by Se treatment; Table S3: Inhibition of mycelial growth of *B. cinerea* by compound treatment with MT+Se; Table S4: Media component; Table S5: The qRT-PCR primers; Table S6: Statistical table of sequencing data results of strawberry fruits samples; Table S7: Up-regulated resistance genes in response to MT+Se treatment; Figure S1: Statistical overview and Venn diagrams of differentially expressed genes (DEGs) across each comparison group; Figure S2: Gene Ontology (GO) enrichment analysis of DEGs for each comparison group; Figure S3: Bubble plot of KEGG enrichment analysis of differential genes in each comparison group; Figure S4: Peak plots of mass spectra of QC samples (A), correlation analysis of QC samples (B), and correlation analysis of fruit samples (C); Figure S5: Differential metabolite statistics results in histograms.
