# Supplementary material for: Melatonin and Selenium Enhance Strawberry Resistance to Botrytis cinerea via Integrated Transcriptome–Metabolome Analysis
Source: Foods. 2026 Sep 17;15(18):3291. doi: 10.3390/foods15183291 (PMC13606195; doi:10.3390/foods15183291)
Supplement: Supplementary file 1 [file foods-15-03291-s001.zip › foods-4571668-supplementary.pdf]

Supplementary Information

Melatonin and selenium enhance strawberry resistance to *Botrytis cinerea* via integrated transcriptome–metabolome analysis

S1. Reagents

Most of the analytical grade reagents used in research, such as sodium selenite, melatonin, disodium hydrogen phosphate, dimethyl phosphate, hydrogen peroxide, thiobarbituric acid, anhydrous ethanol, ethylenediaminetetraacetic acid (EDTA), Coomassie brilliant blue G-250, bovine serum albumin (BSA), p-aminobenzenesulfonic acid, polygalacturonic acid, catechol, L-phenylalanine, ferric chloride, ferrous chloride, and acetone, are commonly purchased from well-known suppliers in China. Sodium selenite is often supplied by Fengchen Group, while melatonin is available from several reliable manufacturers including Qyherb (Xi'an DN Biology), Bestway (Shandong), SCM Pioneering, OctagonChem (Hubei), Nutrakey/Saikang Pharmaceutical (the only Chinese supplier approved for pharmaceutical grade), Wuhan Ability Chemical, and other Xi'an-based bioengineering companies. Disodium hydrogen phosphate is widely produced in China, with common suppliers being Aurora Industry, Hubei Jusheng Technology, Shaanxi Dideu Medichem, and Hefei TNJ Chemical. For the other chemical reagents, researchers typically obtain them from established Chinese laboratory suppliers such as Aladdin (Shanghai Aladdin Biochemical Technology Co., Ltd.) and Sigma-Aldrich China, both of which provide high-purity analytical grade materials that are frequently cited in the literature for biochemical and physiological experiments.

S2. Strawberry Fruit Sample Processing and RNA Extraction

Strawberry fruits at the red-ripening stage, uniform in size and free of visible damage, were harvested. Fruits were surface sterilized by immersion in 2% sodium hypochlorite for 2 min, rinsed twice with sterile distilled water, and air-dried under sterile conditions. The fruits were divided into two experimental groups: the sterile water control group (CK) and the sodium selenite plus melatonin treatment group (MS).

Using sterile toothpicks, ~2 mm deep punctures were made at the equatorial region of each fruit. After allowing wound exudates to air-dry, 10 μL of sterile water (CK) or 10

μL of the sodium selenite–melatonin mixture (MS) was injected into the puncture site. Fruits were left undisturbed until complete absorption, which was designated as 0 h. At this point, both groups were inoculated by injecting 10 μL of a Botrytis cinerea spore suspension (1 × 10^6 conidia/mL) into the same site. Following absorption, fruits were incubated at 22 °C and 80% relative humidity (RH). Disease development was monitored over time.

Samples were collected at two stages: immediately after treatment (0 h) and upon the onset of visible disease symptoms. For each sample, tissues were excised ~2 mm around the inoculation site, cut into uniform pieces, rapidly frozen in liquid nitrogen, and stored at –80 °C for subsequent analyses. Each treatment included three biological replicates.

Sample number	Treatment	Time (h)	Sample number	Treatment	Time (h)
CK-0-1	Control (uninoculated)	0	MS-0-1	Treatment (uninoculated)	0
CK-0-2	Control (uninoculated)	0	MS-0-2	Treatment (uninoculated)	0
CK-0-3	Control (uninoculated)	0	MS-0-3	Treatment (uninoculated)	0
CK-3-1	Control (inoculated)	72	MS-3-1	Treatment (inoculated)	72
CK-3-2	Control (inoculated)	72	MS-3-2	Treatment (inoculated)	72
CK-3-3	Control (inoculated)	72	MS-3-3	Treatment (inoculated)	72

Total RNA was extracted using the Plant Total RNA Isolation Kit with magnetic beads (Tiangen Biotech, Beijing, China), optimized for polysaccharide- and polyphenol-rich tissues. Briefly, frozen strawberry samples were ground into a fine powder in liquid nitrogen using a pre-chilled mortar and pestle. Approximately 20 mg of tissue powder was transferred into a 1.5 mL centrifuge tube containing 450 μL lysis buffer (Buffer SL). After vortexing, the lysate was incubated at room temperature for 5 min and

centrifuged at 12,000 rpm for 5 min. The supernatant (450 µL) was transferred to a new tube, mixed with 400 µL isopropanol and 40 µL magnetic bead suspension (W), and incubated for 5 min to allow RNA binding.

The tubes were placed on a magnetic rack for 3 min, and the supernatant was carefully discarded. Beads were washed sequentially with 900 µL protein-removal buffer (RD, supplemented with ethanol), followed by DNase I digestion (5 µL DNase I + 70 µL RDD buffer, 15 min incubation at room temperature). Beads were subsequently washed twice with RD buffer and twice with wash buffer RW (with ethanol). After air-drying at room temperature for 5 min, RNA was eluted in 100 µL RNase-free water preheated to 60 °C. Eluted RNA was collected after 5 min incubation with intermittent inversion, transferred to a new RNase-free tube, and stored at –80 °C. RNA concentration and purity were assessed using a NanoDrop 2000 spectrophotometer (Thermo Scientific, Waltham, MA, USA).

mRNA Library Preparation and Sequencing

Samples with ⩾1 µg total RNA were used for library construction with the NEBNext Ultra II RNA Library Prep Kit for Illumina (New England Biolabs, Ipswich, MA, USA). Polyadenylated mRNA was enriched using Oligo(dT)-conjugated magnetic beads, and fragmented into ~200 nt pieces using divalent cations at elevated temperature. First-strand cDNA was synthesized using random hexamer primers, followed by second-strand synthesis. The resulting double-stranded cDNA was end-repaired, A-tailed, and ligated with Illumina sequencing adapters. Size selection was performed using AMPure XP beads to enrich cDNA fragments of 400–500 bp, followed by PCR amplification and purification with AMPure XP beads. Library quality was assessed using the Agilent 2100 Bioanalyzer with the Agilent High Sensitivity DNA Kit. Library concentration was determined by fluorescence quantification with Quant-iT PicoGreen (Invitrogen, Carlsbad, CA, USA) using a Quantifluor-ST fluorometer (Promega, Madison, WI, USA), and by qPCR quantification (StepOnePlus Real-Time PCR System, Thermo Scientific). After normalization and pooling of multiplexed libraries, paired-end sequencing (PE150) was performed on an Illumina platform.

S3. Figures

Supplementary Figure S1

Statistical overview and Venn diagrams of differentially expressed genes (DEGs) across each comparison group.

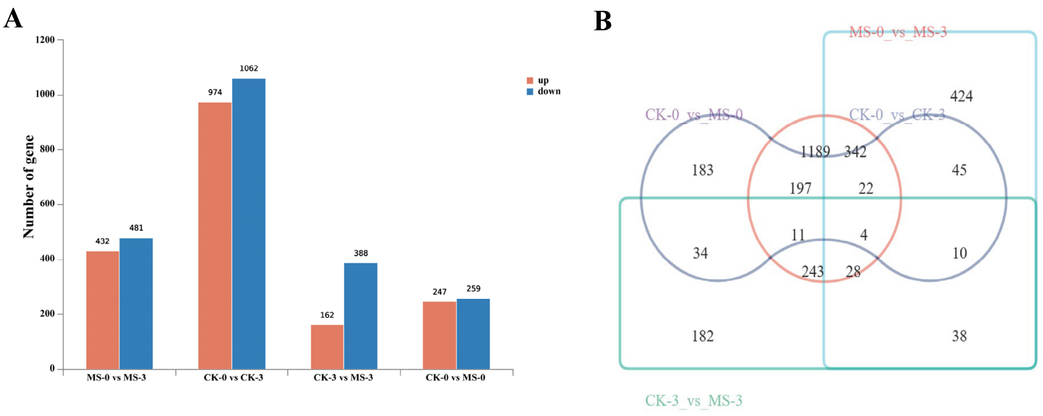

Note: (A) Statistical overview; (B) Venn diagrams.

Supplementary Figure S2

Gene Ontology (GO) enrichment analysis of DEGs for each comparison group.

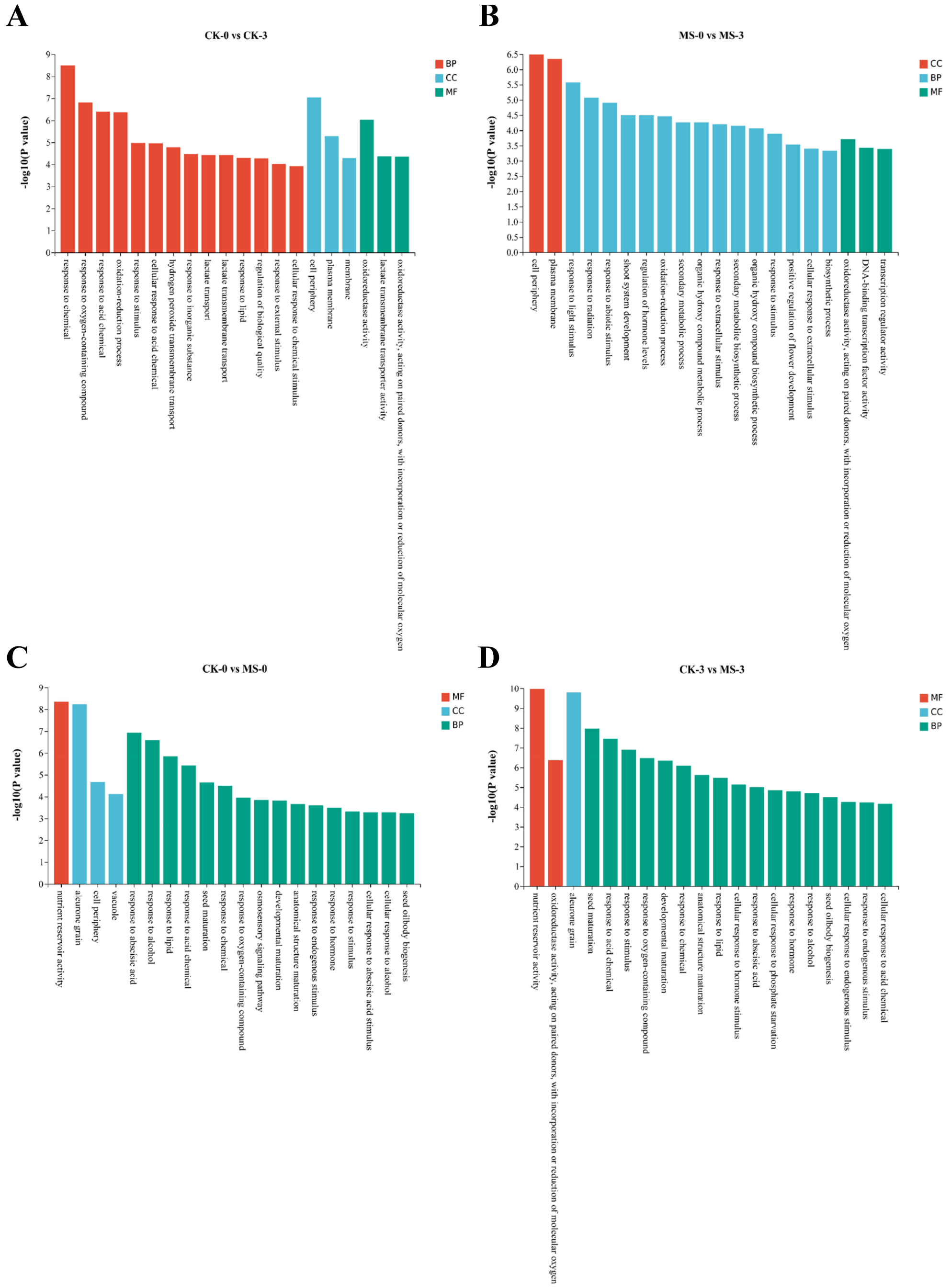

Note: (A) CK-0 vs CK-3; (B) MS-0 vs MS-3; (C) CK-0 vs MS-0; (D) CK-3 vs MS-3.

BP: Biological Processes; CC: Cellular Components; MF: Molecular Functions.

Supplementary Figure S3

Bubble plot of KEGG enrichment analysis of differential genes in each comparison group.

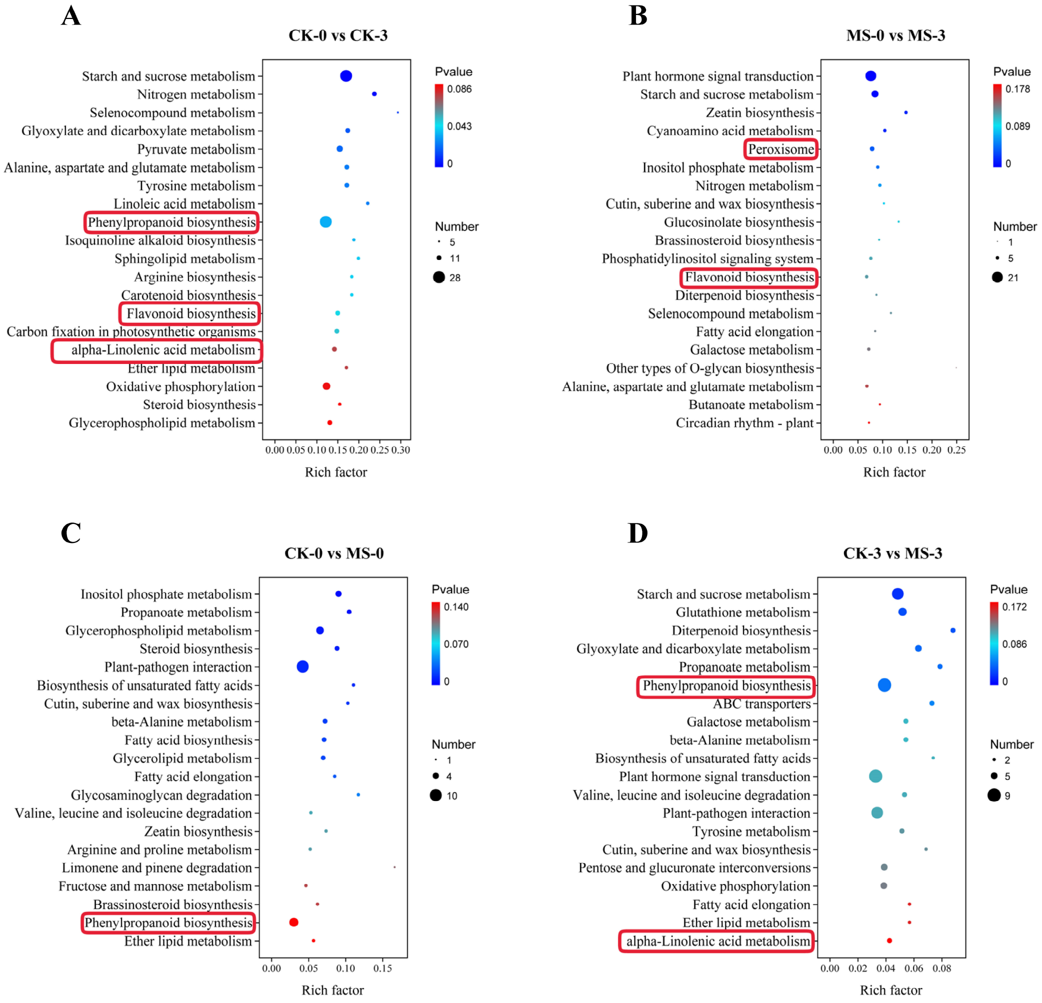

Note: (A) CK-0 vs CK-3; (B) MS-0 vs MS-3; (C) CK-0 vs MS-0; (D) CK-3 vs MS-3.

Supplementary Figure S4

Peak plots of mass spectra of QC samples (A), correlation analysis of QC samples (B), and correlation analysis of fruit samples (C).

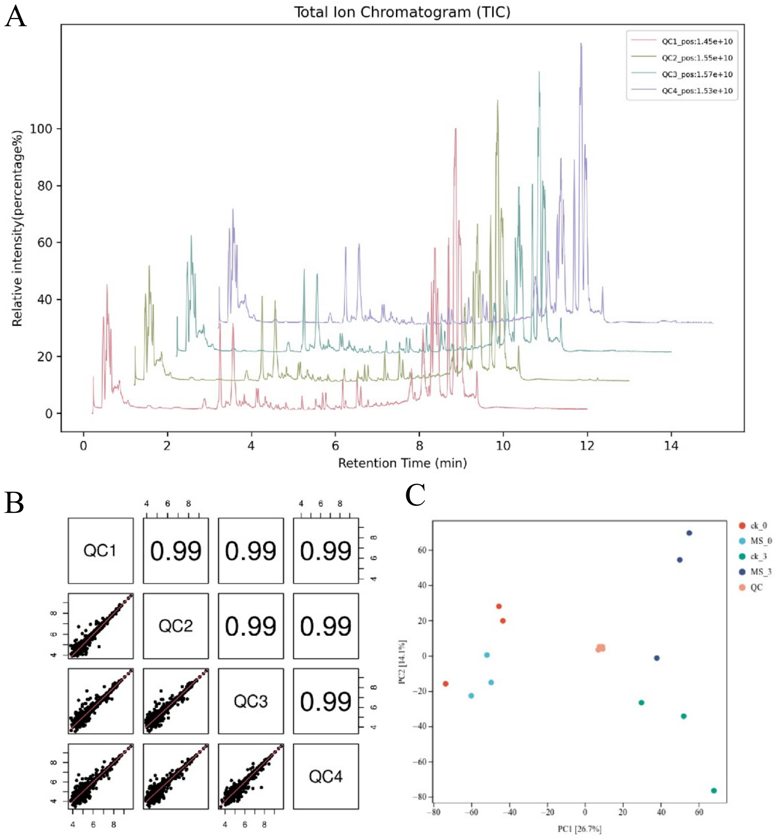

Supplementary Figure S5

Differential metabolite statistics results in histograms.

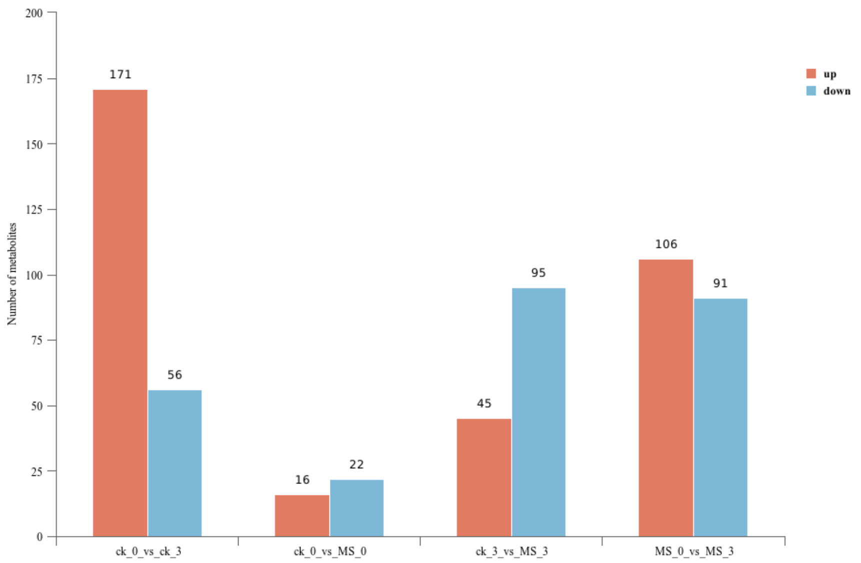

Tables:

Table S1. Inhibition of mycelial growth of *B. cinerea* by melatonin treatment.

Concentration（mmol/L）	Average colony diameter（cm）	Inhibition rate（%）
0	5.92	-
0.01	6.18	-18.39±13.01aA
0.05	5.9	0.38±0.23aA
0.1	5.58	6.51±4.53aA
0.5	5.18	14.18±9.67aA
1	5.55	7.08±4.30aA
1.5	5.48	8.43±4.90aA
2	5.23	13.22±7.93aA

Note: Lowercase English letters indicate significant differences among treatment groups at the P < 0.05 level, and uppercase English letters indicate significant differences among treatment groups at the P < 0.01 level.

Table S2. Inhibition of mycelial growth of *B. cinerea* by Se treatment.

Concentration（mg/L）	Average colony diameter（cm）	Inhibition rate（%）	EC50（mg/L）
0	5.40	-	
20	3.60	38.39±0.10dD	EC_{50}=25
30	2.33	65.54±0.21cC	
40	1.97	72.64±0.14bB	
50	1.27	87.74±0.17aA	

Note: Lowercase English letters indicate significant differences among treatment groups at the $P < 0.05$ level, and uppercase English letters indicate significant differences among treatment groups at the $P < 0.01$ level. The correlation coefficient is 0.98.

Table S3. Inhibition of mycelial growth of *B. cinerea* by compound treatment. with MT+Se

Concentration (mmol/L)	Average colony diameter (cm)	Inhibition rate (%)
CK	6.47±0.04	-
MT	5.18±0.05	14.08±0.09cC
Se	4.00±0.14	42.87±0.16bB
MT+Se	1.05±0.05	93.67±0.26aA

Table S4. Media component.

Culture medium	Main components
Potato Dextrose Agar (PDA)	200 g potatoes, 20 g glucose, 20 g agar, 1 L distilled water
Liquid PDA (PDB)	200 g potatoes, 20 g glucose, 1 L distilled water

Table S5. *The qRT-PCR primers.*

Gene name	Forward primer (5′→3′)	Reverse primer (5′→3′)
Actin	ATTACTGCCTTGGCCCCTAG	TCTGTGCACAATTGAAGGGC
PAL	CTTGCTACCTATCCATTG	TCTTCAGTTCCTCTTCAA
4CL	ATAACTCAAACAGCCTAC	CAACACATCCTTATCACA
ACOX1	ACAAGTTACACGAGAAGGA	GAAGCATCAGCCACAATT
CALM	ATGGAGGACGTGTTCAAGGT	CCCAATTTGATCATCCCCGC
HCT	AGTAGTGATGGAGGAGAG	TAGGTTGTTGAAGTTGAGT
CCR	CAGAGCCTATATGACACT	TCTTGATGGAGTCTTCTT
JOM (Jasmonate-O-methyltransferase)	AGTAGCGAAGACGATAAG	AACAAGGCATCCATCATA
DWF4	AACTTCATCAGTCTCCATA	TCTCCTGCTATCTTCTTG

Table S6. Statistical table of sequencing data results of strawberry fruits. samples

Samples	Raw Reads	Clean Reads	Clean Bases (G)	Raw Q20 (%)	Raw Q30 (%)	Clean GC (%)	Clean Reads Ratio (%)	Total Mapped (%)	Uniquely Mapped (%)
ck_0_1	51184080	50347552	7.57	97.44	95.43	45.75	98.37	82.23	97.11
ck_0_2	40778566	40011612	6.03	97.24	95.02	45.68	98.12	82.24	97.33
ck_0_3	61001314	59911684	9.02	97.34	95.21	45.7	98.21	82.25	97.24
ck_3_1	39454484	38703970	5.83	97.24	95	46.36	98.10	82.26	97.24
ck_3_2	42755444	41997290	6.33	97.35	95.24	45.51	98.23	82.27	97.16
ck_3_3	48478154	47558158	7.17	97.22	94.97	45.65	98.10	82.28	97.19
MS_0_1	52678098	51760496	7.8	97.37	95.27	45.75	98.26	82.29	97.13
MS_0_2	40152172	39365972	5.93	97.22	94.99	45.75	98.04	82.30	97.31
MS_0_3	49696556	48767294	7.34	97.34	95.23	45.88	98.13	82.31	97.21
MS_3_1	47007460	46131624	6.95	97.24	95.03	45.6	98.14	82.32	97.43
MS_3_2	50368222	49466594	7.45	97.26	95.07	45.68	98.21	82.33	97.36
MS_3_3	53709464	52704658	7.94	97.29	95.11	45.52	98.13	82.34	97.25

Note: Raw Q20 (%) percentage of bases with raw sequencing base identification accuracy of 99% or more; Raw Q30 (%) percentage of bases with raw sequencing base identification accuracy of 99.9% or more; Clean GC (%) the percentage of G and C bases in the total bases in the Clean Data obtained after filtering the original data; Clean Reads Ratio (%) the percentage of the total number of Reads of high-quality sequences to the total number of Reads of the original sequencing after filtering; Total Mapped (%) the total number of sequences compared to the reference genome as a percentage of the total number of filtered high-quality sequence Reads; Uniquely Mapped (%) the total number of sequences compared to only one position as a percentage of the total number of sequences compared to the reference genome.

Table S7. Up-regulated resistance genes in response to MT+Se treatment.

NCBI ID	Gene ID	Domain Class	Log_2Fold Change	P value	Description
LOC101302092	FvH4_6g01120.1	KIN	2.337249	0.000287	PREDICTED: serine/threonine-protein kinase At3g07070-like
LOC101305471	FvH4_5g33590.1	RLK	1.039998	0.012161	PREDICTED: receptor-like protein kinase HAIKU2
LOC101306951	FvH4_6g53800.1	N	1.310824	0.016296	PREDICTED: ABC transporter I family member 11, chloroplastic
LOC101315315	FvH4_3g02290.1	CNL	1.319144	0.020637	PREDICTED: putative disease resistance protein RGA3 isoform X1
LOC101314539	FvH4_2g20310.1	TNL	1.189721	0.021841	PREDICTED: TMV resistance protein N-like
LOC101297860	FvH4_3g09660.1	TRAN	1.071877	0.023872	PREDICTED: protein kinase PINOID
LOC101305321	FvH4_7g21500.1	KIN	2.129855	0.035628	PREDICTED: receptor-like protein kinase THESEUS 1
LOC101291301	FvH4_3g31760.1	KIN	1.265288	0.03716	PREDICTED: receptor-like serine/threonine-protein kinase NCRK
LOC101299278	FvH4_3g42190.1	RLK	1.095755	0.037771	PREDICTED: probable LRR receptor-like serine/threonine-protein kinase At4g26540
LOC101299468	FvH4_6g33080.1	KIN	1.173432	0.04712	PREDICTED: inactive protein kinase SELMODRAFT_444075
